# Supplementary material for: Engineering micro oxygen factories to slow tumour progression via hyperoxic microenvironments
Source: Nat Commun. 2022 Aug 2;13:4495. doi: 10.1038/s41467-022-32066-w (PMC9345862; doi:10.1038/s41467-022-32066-w)

Supplementary Information

Supplementary Table 1

List of Terminology and abbreviations

Terminology/Abbreviation	Explanation/Full name
PMC	photosynthesis microcapsule
UCNP	upconversion nanoparticle
NIR	near infrared
MC	microcapsule
PLL	poly-L-lysine
BG11	blue green algal cell growth medium
DMEM	Dulbecco's modified Eagle medium
DCFH	2′,7′ -dichlorodihydroflorescein
DCF	2′,7′ -dichlorofluorescein
MEF	mouse embryonic fibroblasts
NIR-PMC	PMC coupled with NIR treatment
FITC-MAb1	Anti-pimonidazole mouse monoclonal IgG1 antibody conjugated to FITC
HIF-1α	hypoxic-inducible factor-1α
VCAM-1	vascular cell adhesion molecule-1
fLuc-4T1	firefly luciferase-transfected 4T1 cells
BLI	bioluminescence imaging
PD-1	programmed cell death protein 1 (PD-1)
Anti-PD-1	antibody towards mouse PD-1
TAMs	tumor-associated macrophages
OD	optical density

Supplementary Table 2

The primers of genes in real-time quantitative PCR analysis

Gene	Primer
HIF-1α	F 5' GACAGCCTCACCAAACAC 3'
	R 5' CGCTTTCTCTGGGCATTC 3'
BNIP3	F 5' ATACTCAGCCATGATGTC 3'
	R 5' CAACGGTGTGAATGTAAG 3'
VEGF	F 5' CACGCCTACCCACATACATAAC 3'
	R 5' CCAGTCCACAGCAGTCAAATAC 3'
IGF-2	F 5' TTTGCCTTGTGCTGCATTG 3'
	R 5' TAACTTGCCCACGGAGTAATC 3'
MMP-2	F 5' TCTTCAAGGGCACTTATTAC 3'
	R 5' TGAGAAGGCTGATTAACTAC 3'
TGF-α	F 5' CTTCCCGACTTCTACATTC 3'
	R 5' TCCAGATCCATCTCTACTC 3'
GAPDH	F 5' GTCGGAGTGAACGGATTTG 3'
	R 5' ATCTCGCTCCTGGAAGATG 3'

Supplementary Table 3

Information for all using antibodies

Antibodies name	Catalog numbers	Supplier name	Dilutions	Clone numbers for monoclonals	Applicable /validation
Phospho-IκBα (Ser32/36) (5A5) Mouse mAb	Cat. 9246	Cell Signaling Technology	1:1000	Monoclonal 5A5	WB, IP, IHC, ChIP, IF, F; WB
IκBα (L35A5) Mouse mAb (Amino-terminal Antigen)	Cat. 4814	Cell Signaling Technology	1:1000	Monoclonal L35A5	WB, IP, IHC, ChIP, IF, F; WB
Anti-Actin antibody produced in rabbit	Cat. A2066	Sigma-Aldrich	1:5000	polyclonal	ICH, IF, WB; WB
in vivo Mab anti-mouse PD-1 (CD279)	Cat. BE0146	Bioxcell	10 mg/Kg mouse	Monoclonal RMP1-14	WB, ELISA, IP, IHC, ChIP, IF; SDS-PAGE
Mouse Anti-AKT antibody	Cat. bsm-33278M	Bioss	1:1000	Monoclonal 10D8	WB, ELISA, ICC; WB
PI3K p85 alpha Antibody	Cat. AF6241	Affinity Biosciences	1:500	polyclonal	WB, IHC, IF/ICC, ELISA; WB
Phospho-PI3K p85 (Tyr458) [Tyr467]/p55 (Tyr199) Antibody	Cat. AF3242	Affinity Biosciences	1:500	polyclonal	WB, IHC, IF/ICC, ELISA; WB
Anti-phospho-AKT	Cat. Ab38449	Abcam	1:1000	polyclonal	WB, IHC-P; WB
Rabbit monoclonal [EPR390(N)] to mTOR	Cat. Ab134903	Abcam	1:10000	Monoclonal EPR390(N)	WB; Knockout validated
Rabbit monoclonal [EPR426(2)] to mTOR (phospho S2448)	Cat. Ab109268	Abcam	1:10000	monoclonal [EPR426(2)]	WB, IHC-P, Dot blot, IHC-Fr; Knockout validated
Rabbit monoclonal [Y72] to ERK1	Cat. Ab32537	Abcam	1:1000	Monoclonal [Y72]	WB, IP, IHC-P, Flow Cyt (Intra), ICC/IF; Knockout validated
Rabbit monoclonal [EPR18444] to ERK1 (phospho T202) + ERK2 (phospho T185)	Cat. Ab214036	Abcam	1:1000	monoclonal [EPR18444]	Dot blot, IHC-P, WB, ICC/IF, IP; WB
FITC Rat Anti-Mouse IFN-γ	Cat. 554411	BD Pharmingen	5 μL/test	Monoclonal XMG1.2	IF, FC; verified using the recombinant cytokine blocking and unlabelled antibody blocking specificity control
Purified anti-mouse CD28 Antibody	Cat. 102102	Biolegend	1 μg/mL	Monoclonal 37.51	FC, IP, IHC-F, Costim; Quality tested by intracellular immunofluorescent staining with flow cytometric analysis
Purified anti-mouse CD3ε Antibody	Cat. 100302	Biolegend	1μL/1×10^6 cells	Monoclonal 145-2C11	FC, IHC-F, IP, WB, ICC; Validated by IHC-F

Supplementary Table 4

Blood test of PMC treated animals by subcutaneous injection

Parameter	PMC 1d	PMC 3d	PMC 8d	Range of Healthy Control
WBC	6.3	8.5	6.7	3.2 – 11.0$\times10^9$/L
Lymph#	5.1	6.2	5.1	2.4 – 8.9 $\times10^9$/L
Mon#	0.1	0.2	0.1	0.0 – 0.2 $\times10^9$/L
Gran#	1.1	2.1 ↑	1.5	0.6 – 1.9 $\times10^9$/L
Lymph%	80.3	72.9	75.6	71.0 – 80.8 %
Mon%	1.6	2.7 ↑	2.3	1.6 – 2.6 %
Gran%	18.1	24.4	22.1	16.9 – 26.8 %
RBC	11.9	10.4	11.2	7.9 – 12.6 $\times10^{12}$/L
HGB	179	161	158	116 – 179 g/L
HCT	56.9	48.6	51.4	36.2 – 57.7 %
MCV	47.7	46.7	45.9	44.4 – 49.5 fL
MCHC	314	331	307	303 – 334 g/L
RDW	14.6	12.5	14.1	12.5 – 15.9 %
PLT	880 ↑	794	785	617 – 876 $\times10^9$/L
MPV	6.1	6.4	5.6	5.6 – 7.0 fL

Supplementary Table 5

Blood test of PMC treated animals by intraperitoneal injection

Parameter	PMC 1d	PMC 3d	PMC 8d	Range of Healthy Control
WBC	4.5	6.5	5.5	3.2 – 11.0×10^9/L
Lymph#	3.1	4.9	4.0	2.4 – 8.9 ×10^9/L
Mon#	0.1	0.1	0.1	0.0 – 0.2 ×10^9/L
Gran#	1.3	1.5	1.4	0.6 – 1.9 ×10^9/L
Lymph%	68.0 ↓	74.8	71.8	71.0 – 80.8 %
Mon%	2.4	2.6	2.0	1.6 – 2.6 %
Gran%	29.6 ↑	22.6	26.2	16.9 – 26.8 %
RBC	11.1	10.4	11.8	7.9 – 12.6 ×10^{12}/L
HGB	159	155	161	116 – 179 g/L
HCT	51.3	46.8	51.6	36.2 – 57.7 %
MCV	46.3	45.1	43.8	44.4 – 49.5 fL
MCHC	309	331	312	303 – 334 g/L
RDW	14.9	13.0	14.8	12.5 – 15.9 %
PLT	882 ↑	813	652	617 – 876 ×10^9/L
MPV	6.1	6.7	6.6	5.6 – 7.0 fL

Supplementary Table 6

Cell line identification by STR analysis

**STR loci**	**HeLa in our study**			**HeLa in databases**		
	**Allele1**	**Allele2**	**Allele3**	**Allele1**	**Allele2**	**Allele3**
D19S433	13	14				
D5S818	11	12		11	12	
D21S11	23	27	28			
D18S51	16	16				
D6S1043	18	18				
AMEL	X	X		X	X	
D3S1358	15	18				
D13S317	12	13.3		12	13.3	
D7S820	8	12		8	12	
D16S539	9	10		9	10	
CSF1PO	9	10		9	10	
PentaD	8	15				
D2S441	10	11				
vWA	16	18		16	18	
D8S1179	12	13				
TPOX	8	12		8	12	
PentaE	7	17				
TH01	7	7		7	7	
D12S391	20	25				
D2S1338	17	17				
FGA	18	21				

Note: DSMZ Tools were used for cell line comparison, containing STR data from ATCC, DSMZ, JCRB, CLASTR and RIKEN databases. The authentication was last performed at May 27th 2022 by Sangon Biotech (Shanghai, China).

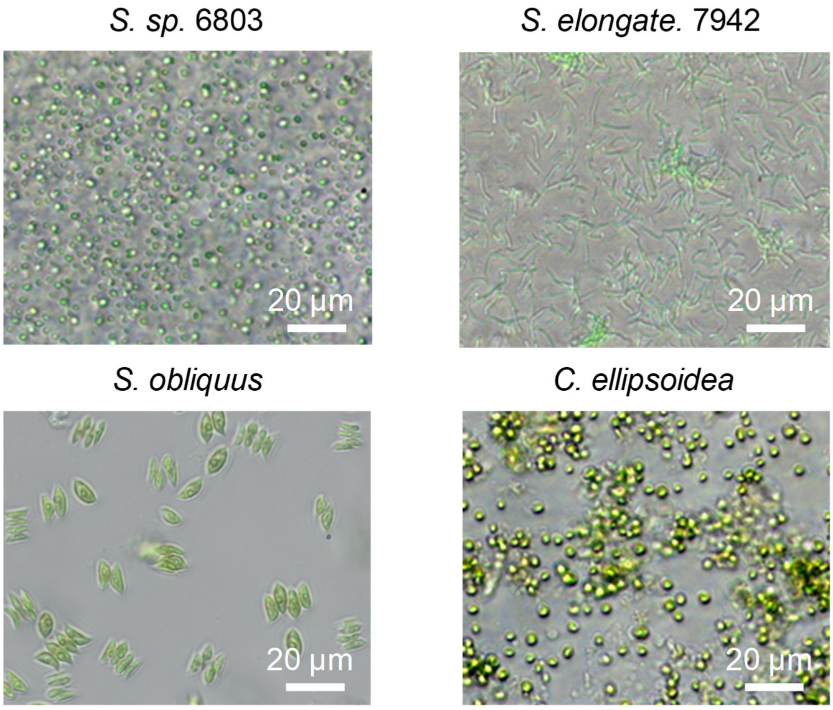

Supplementary Figure 1. Microscopy images of algae

The photographs of *S. sp.* 6803, *S. elongates.* 7942, *S. obliquus* and *C. ellipsoidea* were obtained using a microscope imaging system (Olympus CKX53, Japan) by 40× objective lens.

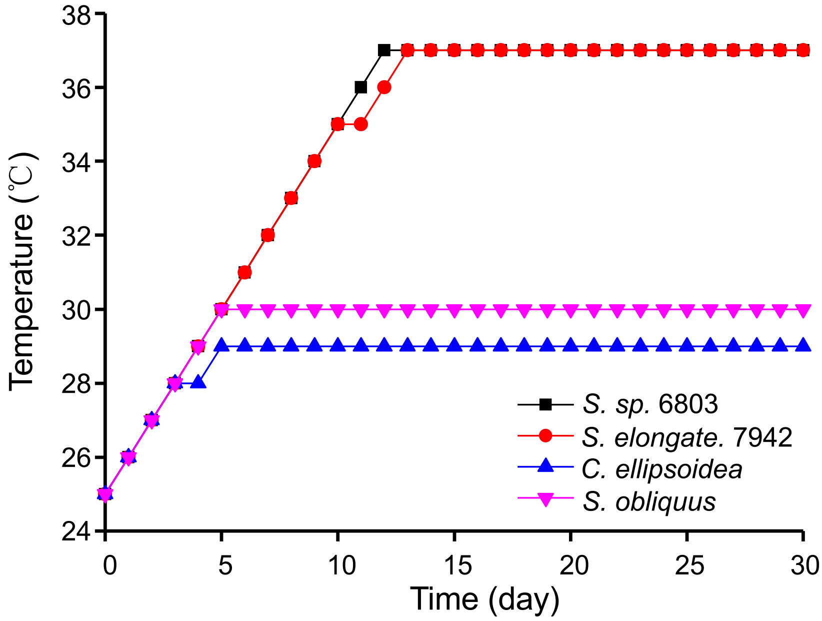

Supplementary Figure 2. Acclimatization of algal strains by stepwise alternation of temperature

S. sp. 6803, S. elongates. 7942, S. obliquus and *C. ellipsoidea* were seeded in BG11 medium at 1.0×10^8 cells/mL, cultured for 30 d from 25 to 37°C. Then the cells were collected and resuspended in 96 well plates at 100 μL to measure their optical density value at 660 nm by microplate reader (n = 3).

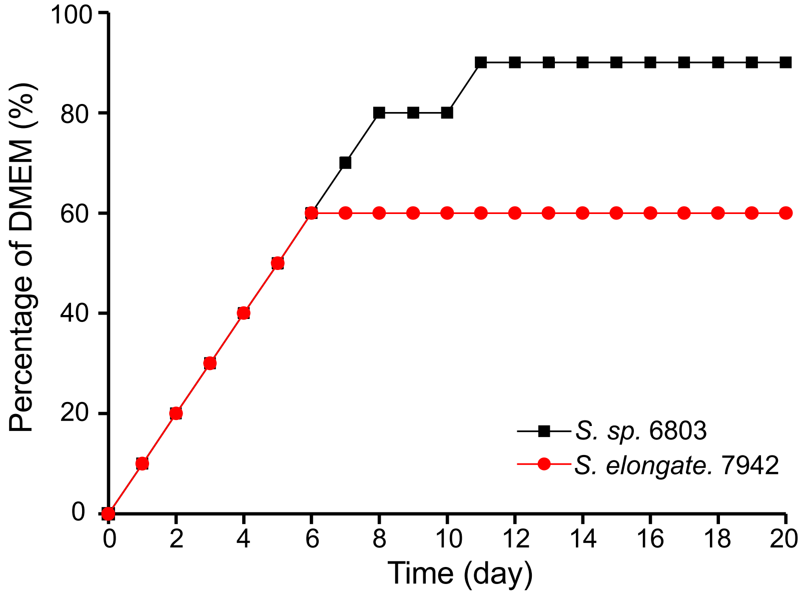

Supplementary Figure 3. Acclimatization of algal strains by stepwise alternation of culture medium

S. sp. 6803, *S. elongates.* 7942 were seeded in BG11 medium at 1.0×10^8 cells/mL by stepwise changing medium composition from BG11 to DMEM cultured. Then the cells were collected and resuspended in 96 well plates at 100 µL to measure their optical density value at 660 nm by microplate reader (n = 3).

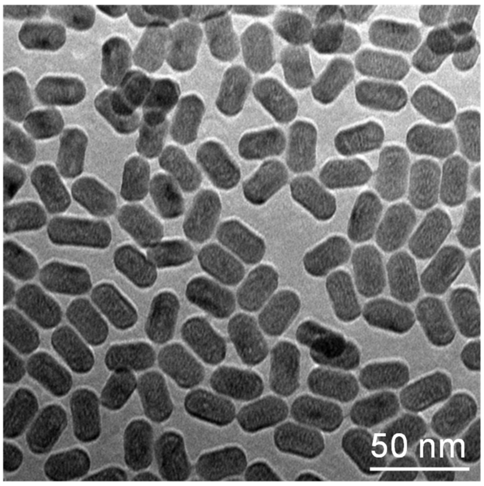

Supplementary Figure 4. TEM images of UCNPs

The nanoparticles were dispersed in hexane at 2 mg/mL and then dropped onto an ultra-thin copper film to visualize their morphology by HR-TEM (FEI TecnaiG2F20 Field emission transmission electron microscope) at 200 kV.

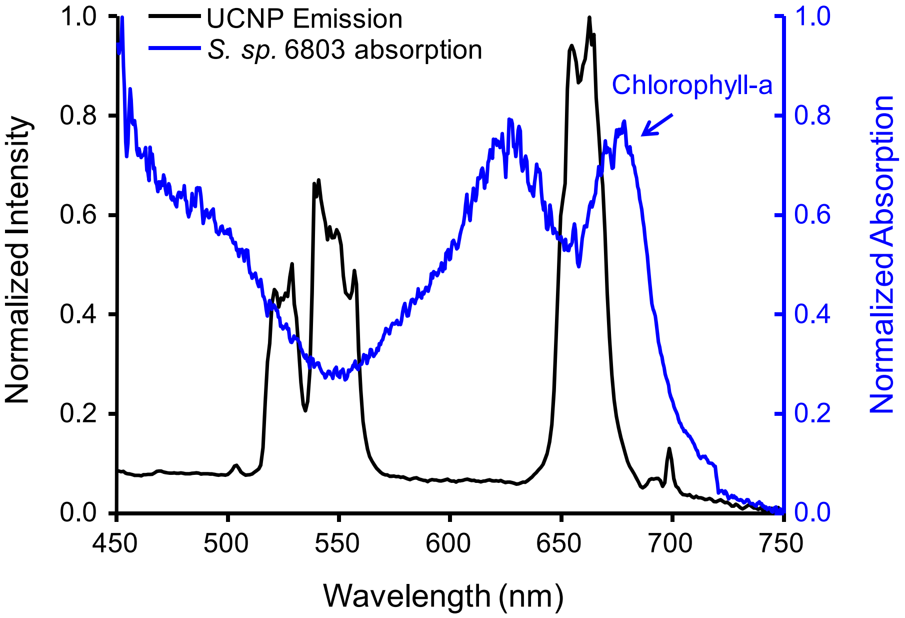

Supplementary Figure 5. Absorption spectra of Chlorophyll-α and emission spectra of UCNPs

S. sp. 6803 was dispersed in water at 1.0×10^8 cells/mL to examine their absorption spectrum (blue line) by UV-Vis spectrophotometer. UCNPs were dissolved in water at 2 mg/mL to examine their emission spectra (black line) by up-conversion spectrophotometer (Edingbour NanoSpectralyzer fluorimetric analyzer with λ_{ex} = 808 nm).

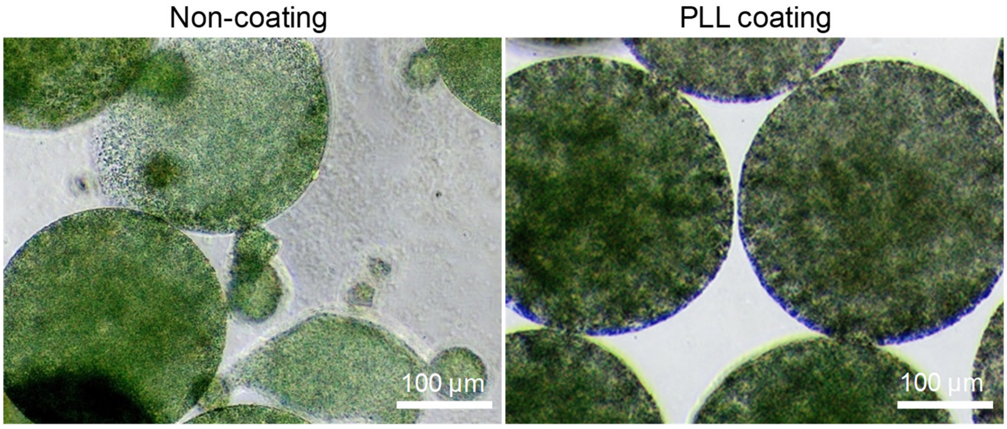

Supplementary Figure 6. Representative images of constructed PMCs with or without PLL coating

Coated and uncoated e-*S. sp.* 6803@MCs were cultured in DMEM for 7 d. Then microcapsules were collected for visualization by microscopy.

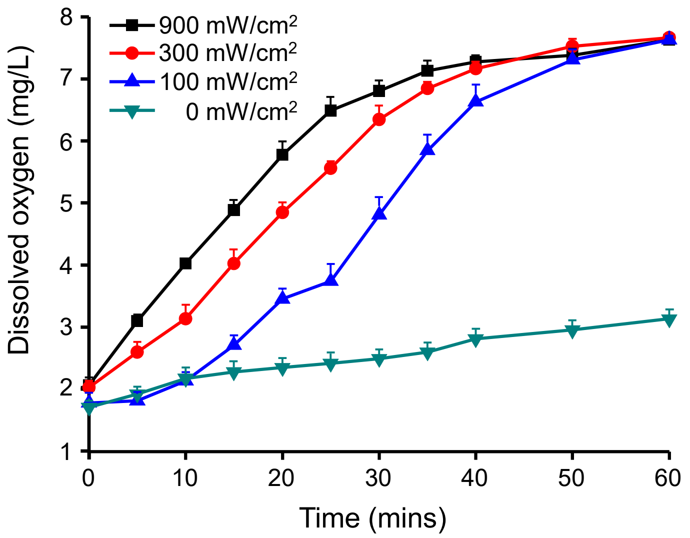

Supplementary Figure 7. Quantification of oxygenation of PMCs at different NIR radiation Impacts of NIR radiation on O_2 synthesis by PMCs. PMCs at 3600/mL were exposed to 100, 300 and 900 mW/cm^2 NIR radiations for O_2 detections. Data are presented as means ± SD, n = 3 independent experiments.

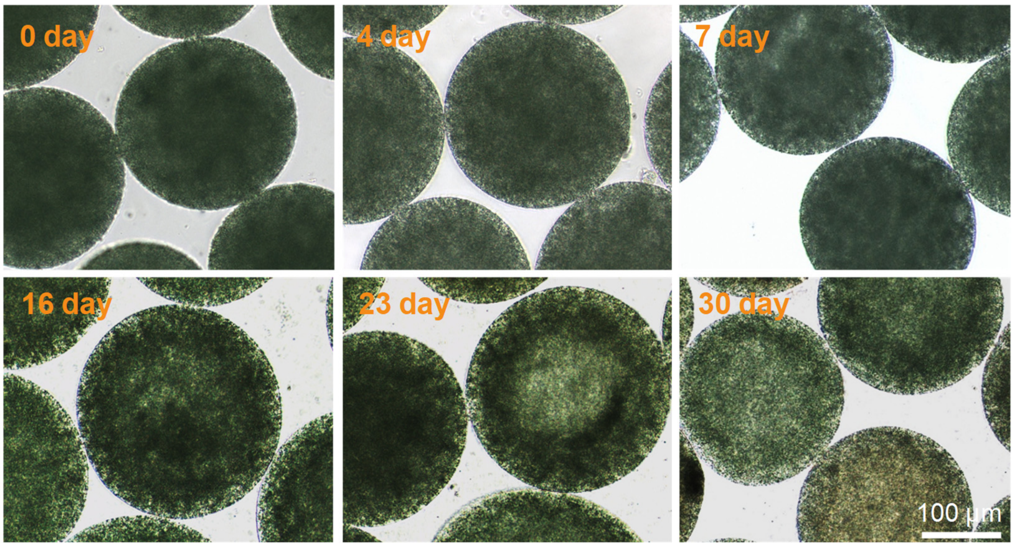

Supplementary Figure 8. Representative images of PMCs cultured in physiological condition

The PMCs were cultured in DMEM media at 3600/mL to capture images at 0, 4, 7, 16, 23 and 30 d.

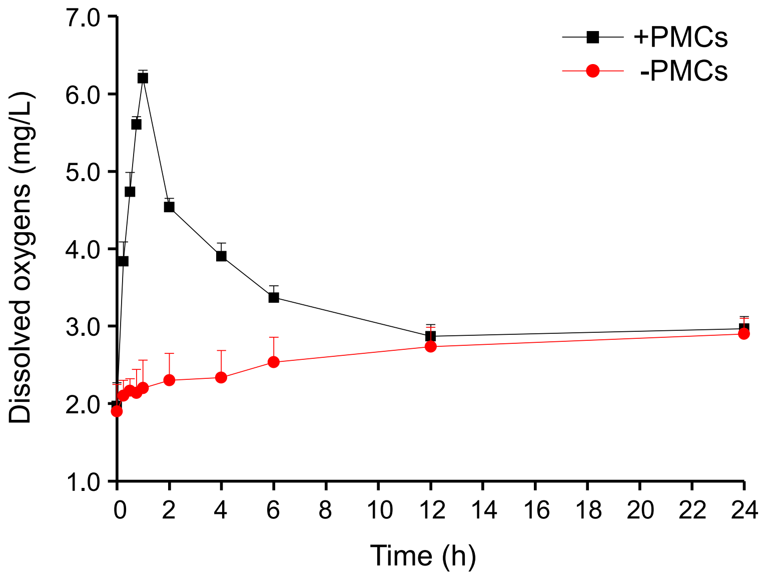

Supplementary Figure 9. Quantification of dissolved oxygen in cell culture media

PMCs at 3600/mL in 10 mL oxygen-free DMEM were exposed to 900 mW/cm^2 980 nm NIR for 60 min. The medium was placed in the dark at 37 °C. Data are presented as means ± SD, n = 3 independent experiments. A portable dissolved oxygen meter (JPB-70A oxygen sensor, Qiwei Instrument Co., Ltd. Hangzhou, China) was used to detect the dissolved oxygen.

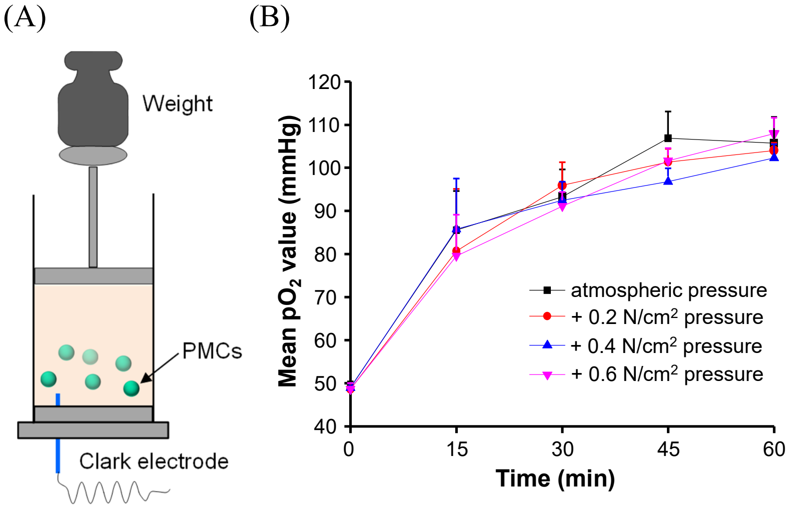

Supplementary Figure 10. Impacts of pressures on oxygenation capability of PMCs

(A) Schematic image of constructed device to detect pO_2 at different pressures and **(B)** pO_2 in DMEM under extra pressures at 0-0.6 N/cm^2. The pO_2 values were recorded at 0, 15, 30, 45 and 60 min post-NIR exposure. Three biological independent replicates were performed. Data were presented as means ± SD, n = 3 independent experiments.

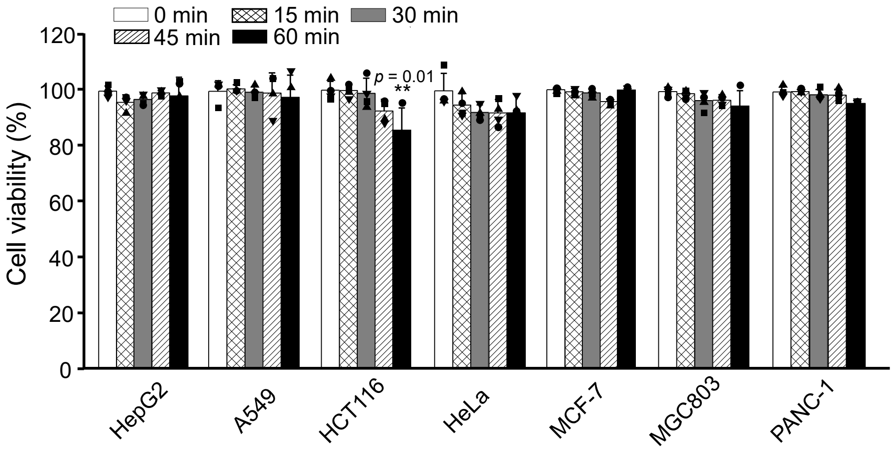

Supplementary Figure 11. Impacts of NIR radiation on cell viability

HepG2, A549, HCT116, HeLa, MCF-7, MGC-803, PANC-1 cells at 5000 cells/well in 96-well plates were exposed to 300 mW/cm^2 980 nm laser for 0, 15, 30, 45 and 60 mins. The cell viability was examined by MTS assay (n = 4). Data are presented as means ± SD. $^{**}p < 0.01$ compared to the control cells without NIR exposure by two-tailed Student t-test.

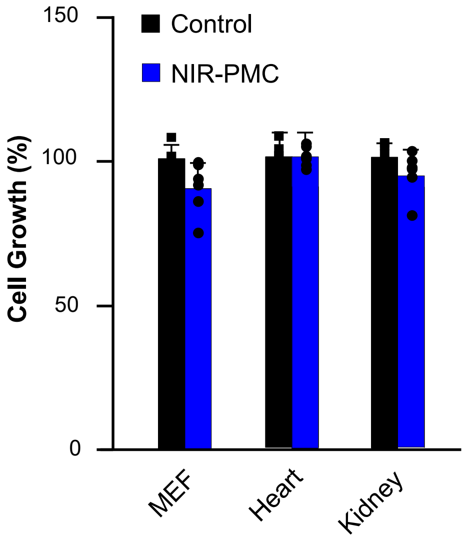

Supplementary Figure 12. Impacts of NIR-PMCs on non-cancer cell proliferation

Cell proliferation assessment. MEF, primary heart and kidney cells incubated with or without PMCs (25 µL, 3.6×10^{4}/mL) were exposed to 300 mW/cm^2 NIR radiation for three intervals. After 24 h, the cell viability was examined by MTS assay (n = 6). Data are presented as means ± SD.

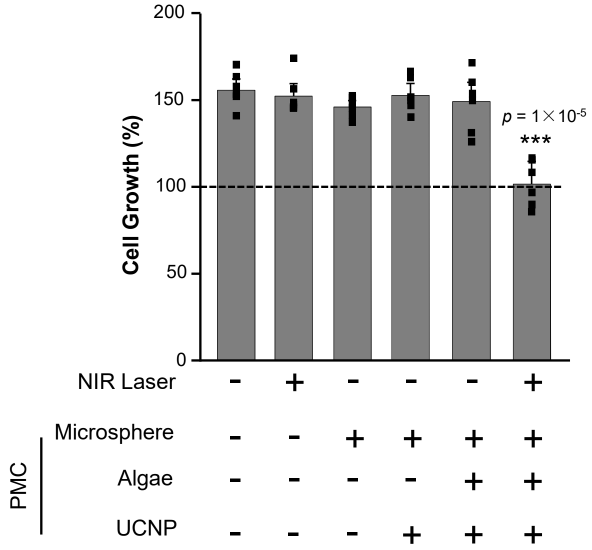

Supplementary Figure 13. Impacts of integrates in PMCs on MCF-7 cell proliferation

MCF-7 cells were incubated with MCs, UCNP@MCs, e-*Synechocystis*@MCs and PMCs in the presence or absence of NIR radiations for cell viability assessment (n = 6). Data are presented as mean ± SD. ***$p < 0.001$ compared to control group by two-tailed Student t-test.

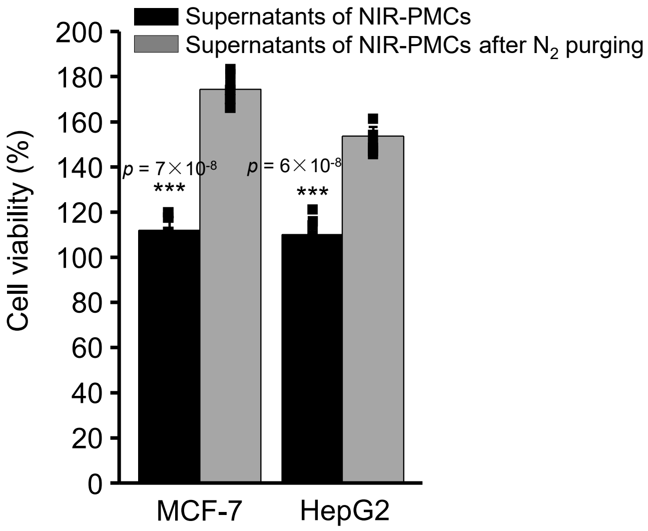

Supplementary Figure 14. Impacts of dissolved oxygens on cancer cell proliferation

Hyperoxic supernatants were collected in PMCs (3600/mL) cultured in DMEM and exposed to three intervals of NIR radiation. N_2 purging were exploited to remove dissolved oxygens in the collected supernatants of NIR-PMCs. MCF-7 and HepG2 cells were exposed to the hyperoxic media before and after N_2 purging for 24 h. The cell viability was examined by MTS assay (n = 6). Data are presented as means ± SD. ***$p < 0.001$ compared to cells cultured in hyperoxic media after N_2 purging by two-tailed Student t-test.

(A)

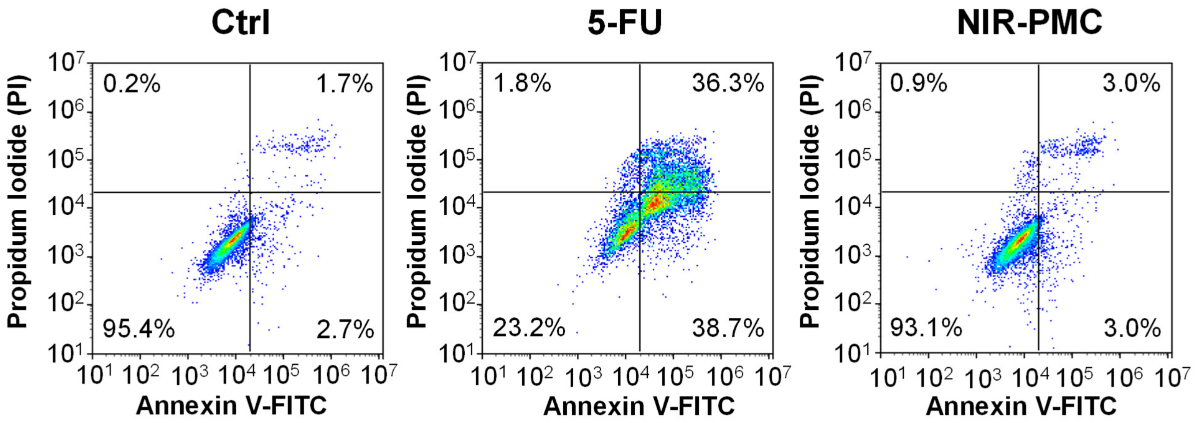

(B)

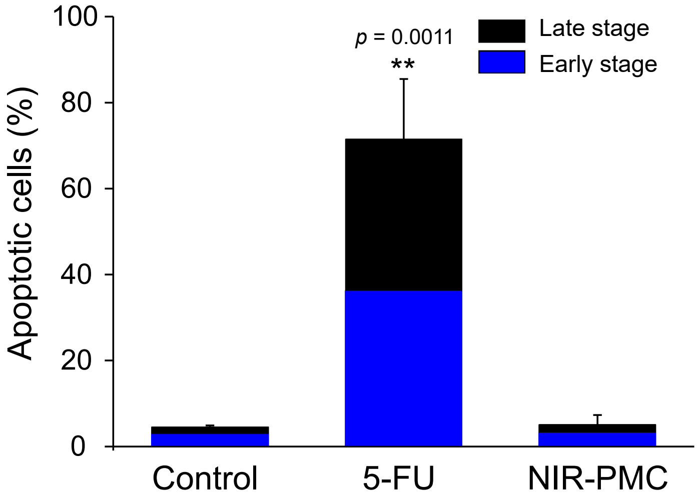

Supplementary Figure 15. Detection of apoptotic cells by flow cytometry

(A) Representative images and **(B)** quantifications of apoptotic cells by flow cytometry analysis. HepG2 cells seeded in six-well plates (5×10^5 cells/well) were exposed to PMCs at 3600/mL with 300 mW/cm^2 NIR for three intervals or 500 µg/mL 5-FU. After 24 h incubation, the cells were stained by Annexin V-FITC and PI for flow cytometry analysis. Three biological independent replicates were performed. Data are presented as means ± SD. n = 3 biologically independent cells, $^{**}p < 0.01$ compared to Control group by two-tailed Student t-test.

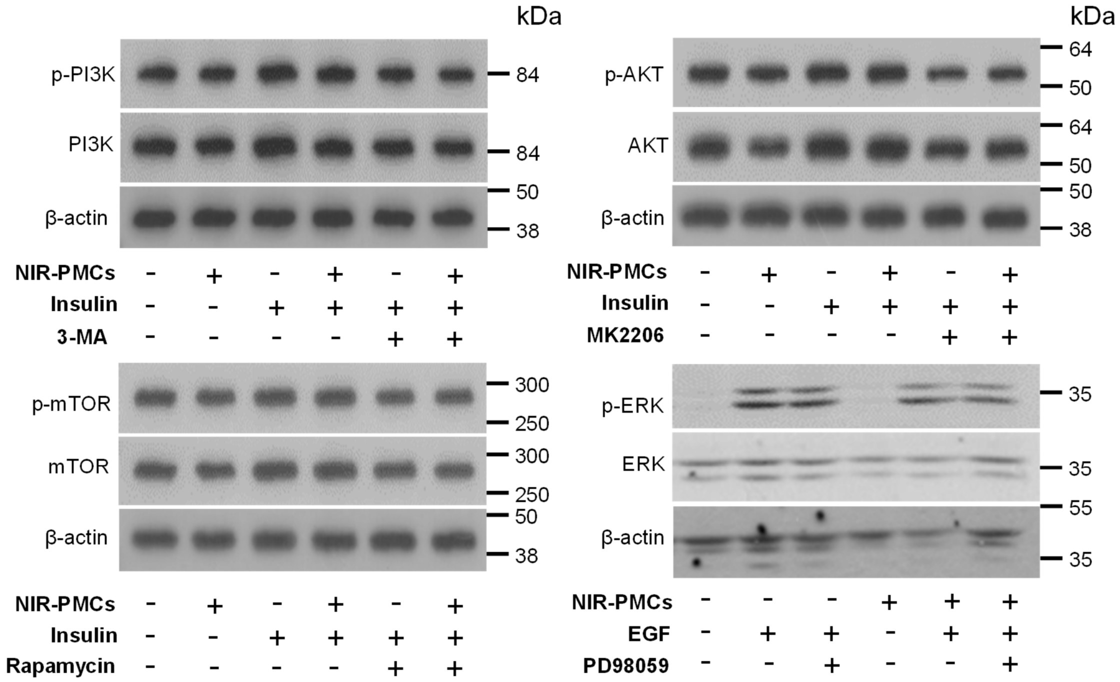

Supplementary Figure 16. Western blotting imaging of PI3K, AKT, mTOR and ERK in HepG2 cells

HepG2 cells incubated with or without PMCs were exposed to 300 mW/cm^2 NIR radiation for three intervals. Then the cells were treated with activators: insulin (100 nM, 15 min) or EGF (200 ng/mL, 10 min), followed by incubation with inhibitors: 3-MA (2 mM, 1 h), MK-2206 (4 µM, 4 h), rapamycin (100 nM, 4 h) or PD98059 (100 µM, 2 h). Cell lysates were collected and subjected to Western blotting analysis of PI3K, AKT, mTOR and ERK.

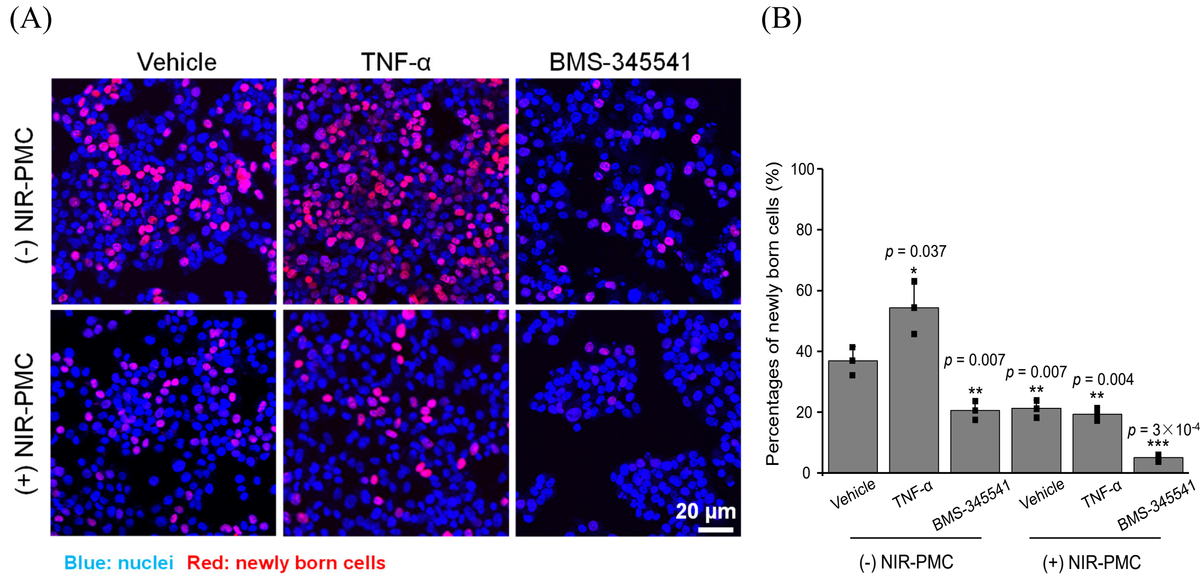

Supplementary Figure 17. Staining of new daughter HepG2 cells after inhibition or activation of NF-κB

(A) Confocal visualization and **(B)** percentages of new daughter cancer cells in different treatments. HepG2 cells (5×10^5 cells/well) were seeded in six-well plates and incubated with/without PMCs at 3600/mL under 300 mW/cm^2 NIR radiation for three intervals. The cells were exposed to BMS (40 μM) for 16 h, or TNF-α (1 ng/mL) for 12 h. After that, HepG2 cells were stained by BeyoClick-iT® EdU-594 assay kit for confocal imaging (scale bar: 20 μm). The percentages of new daughter cells were calculated in six randomly selected confocal images (n = 3 biologically independent confocal images). Data are presented as means ± SD. ***$p < 0.001$, **$p < 0.01$ and *$p < 0.05$ compared to Control group by two-tailed Student t-test.

(A)

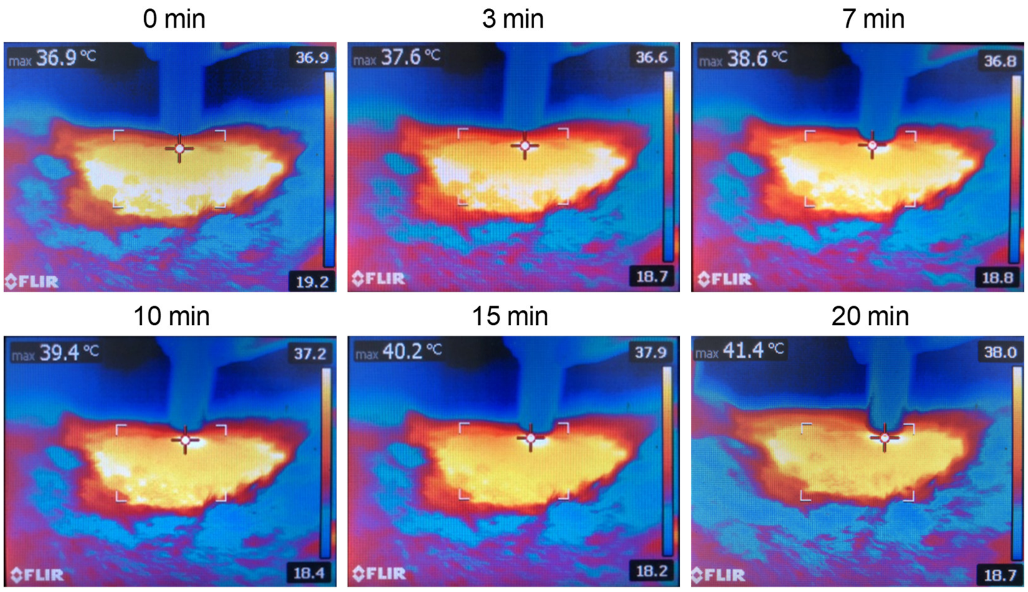

(B)

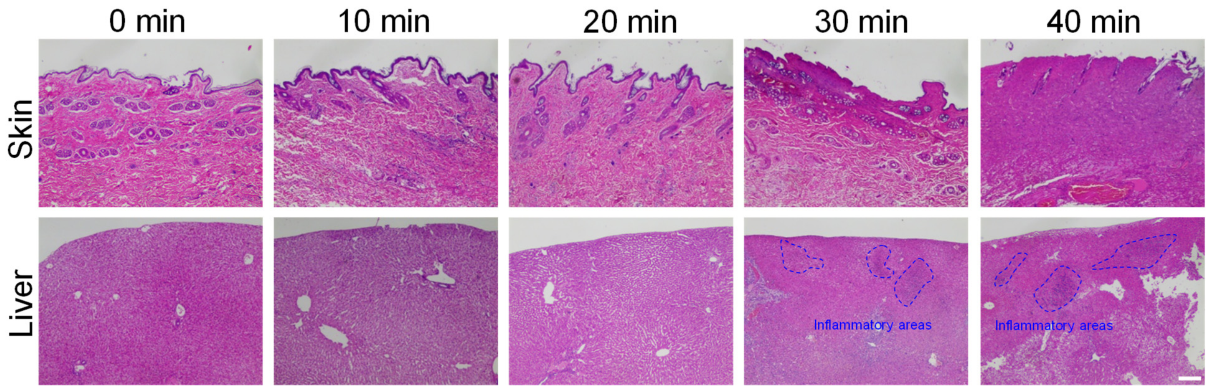

Supplementary Figure 18. Impacts of NIR radiations on rabbit skin and liver

(A) Thermal accumulations on skins and **(B)** H&E staining images of skin and liver in rabbits exposed to 900 mW/cm^2 NIR radiations for 0-40 min. The temperature of rabbit skins exposed to NIR radiations was examined by an infrared thermal imager (FLIR E5, USA). The animals received NIR radiations were sacrificed to collect skin and liver. The tissue samples were fixed in 10% formalin for H&E staining. The inflammatory areas were indicated by dashed blue lines. Scale bars represent 500 μm.

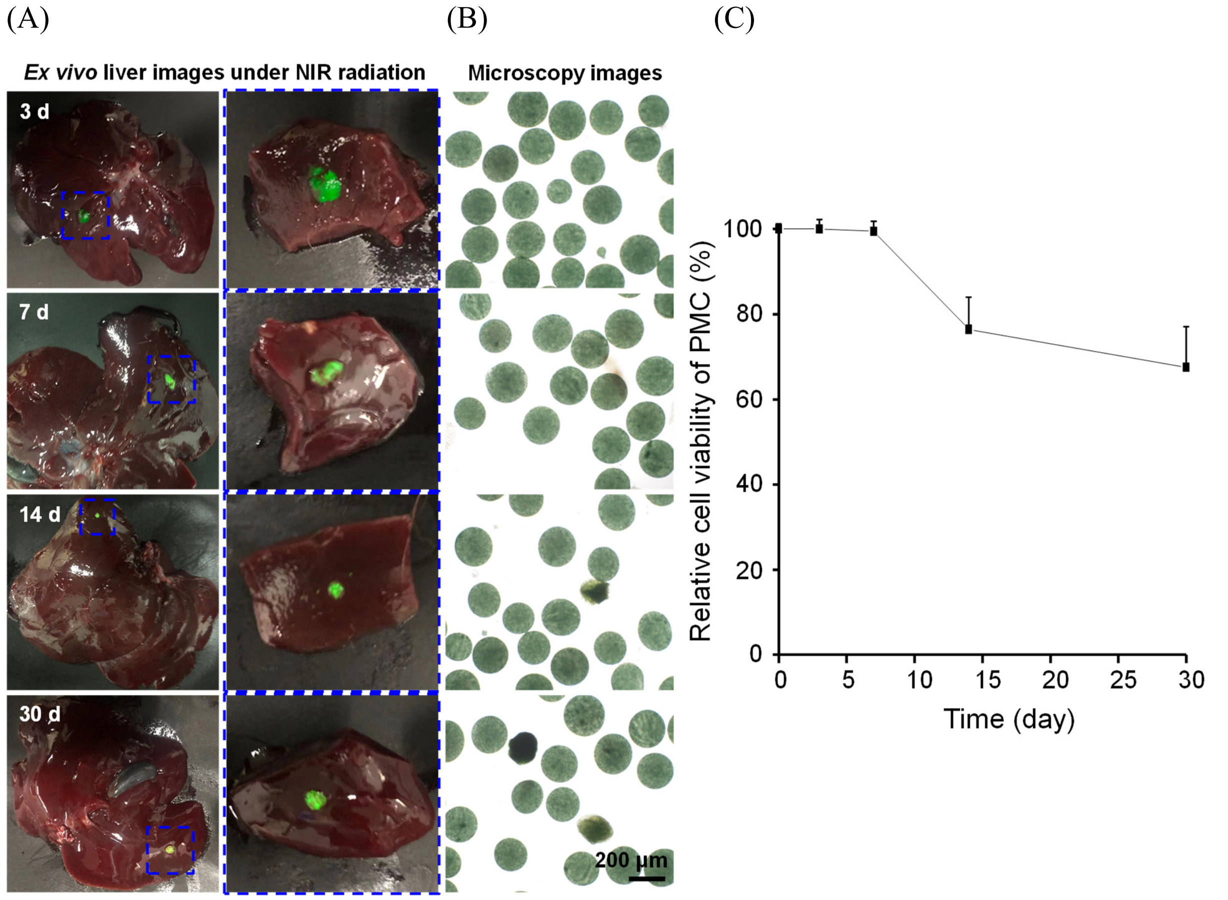

Supplementary Figure 19. Stability of PMCs *in vivo*

(A) The *Ex vivo* liver images under NIR radiation, **(B)** microscopy images and **(C)** viability of extracted PMCs in animal livers at the 3rd, 7th, 14th and 30th d post-PMC injection. PMCs at 36000/mL (500 μL) were injected in rabbit livers and further exposed to 980 nm NIR radiation for 60 min/day. The PMCs were collected from livers of treated animals, suspended in water and examine their optical density at 660 nm by Microplate Reader (n = 3). Data are presented as means ± SD. The numbers of extracted PMCs were determined by inverted microscope (Olympus CKX53). The relative cell activity was assessed by the following equation:

$$\text{Cell viability } \% = \frac{A_i/N_i}{A_0/N_0} \times 100\%$$

where A_i and A_0 are the absorbances of PMC suspensions at the 0 or i^{th} d post PMC injection, respectively; N_i and N_0 are the numbers of PMCs extracted from animal livers at the 0 and i^{th} d post PMC inject, respectively.

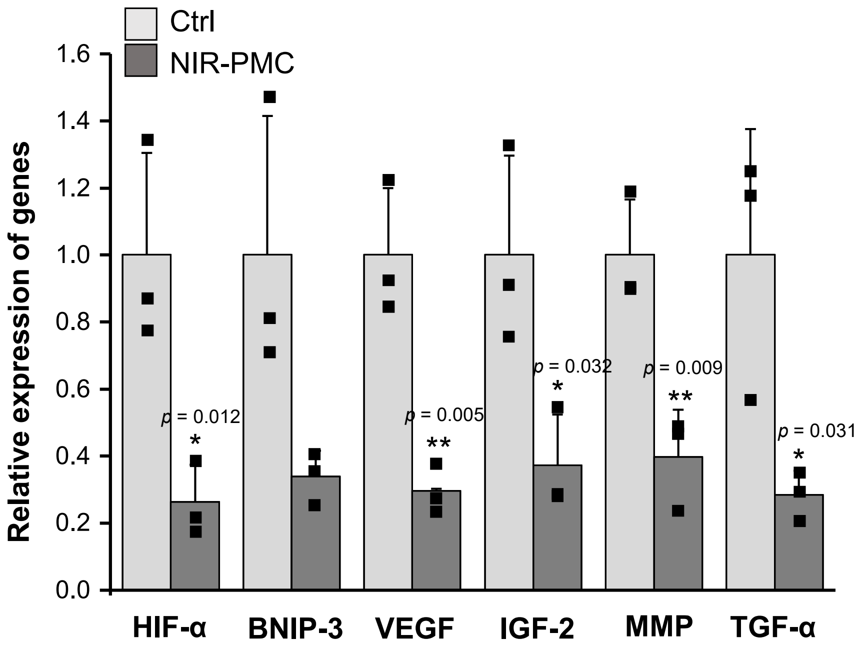

Supplementary Figure 20. The expression of HIF-α and its targets by Real-time quantitative PCR analysis

The total RNA was isolated from the tumour tissues collected from the NIR-PMC treatment or control groups (n = 3 independent rabbits). The resulting cDNA samples were converted by reverse transcriptase and analyzed by real-time PCR with an SYBR Green assay kit. The mRNA levels of each gene were normalized to that of GAPDH. Data are presented as means ± SD. *$p < 0.05$ and **$p < 0.01$ compared to Ctrl by two-tailed Student t-test.

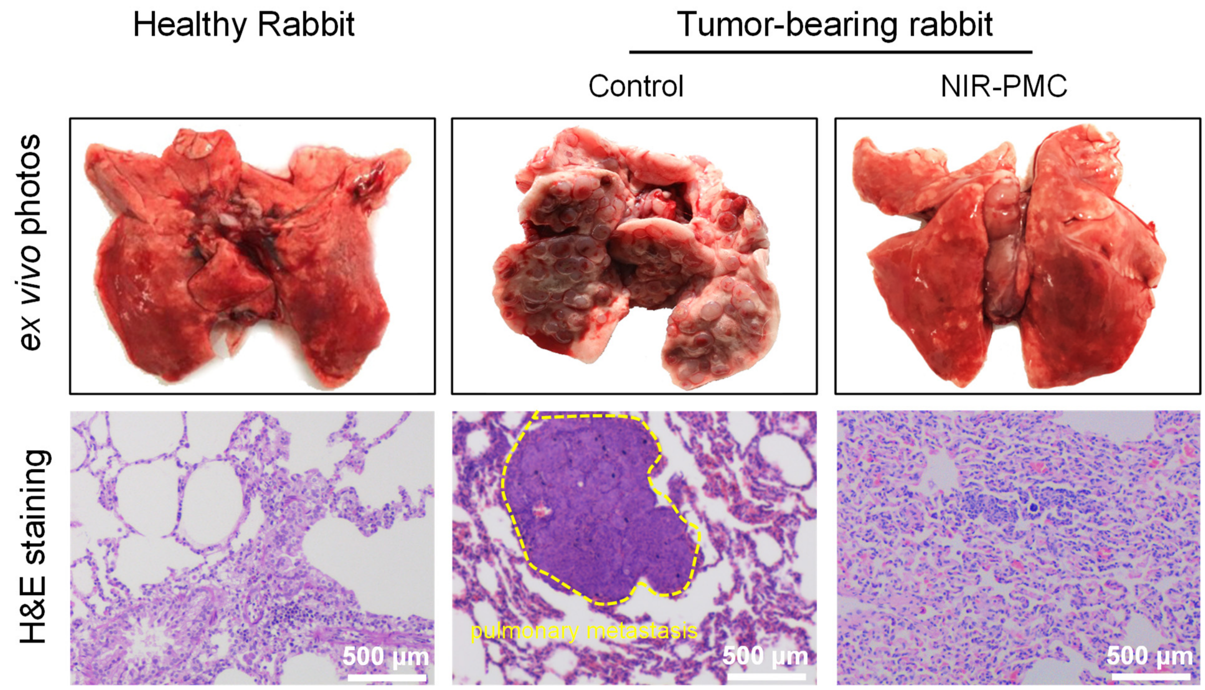

Supplementary Figure 21. *Ex vivo* photos and H&E staining images of pulmonary metastasis

The animals received different treatments were sacrificed on the 28th d for visualization of metastasis nodules in animal lungs. The *ex vivo* images were captured by camera (Canon ZHS2402, Japan). The tissue samples were fixed in 10% formalin for H&E staining. The yellow outlined area indicates pulmonary metastasis.

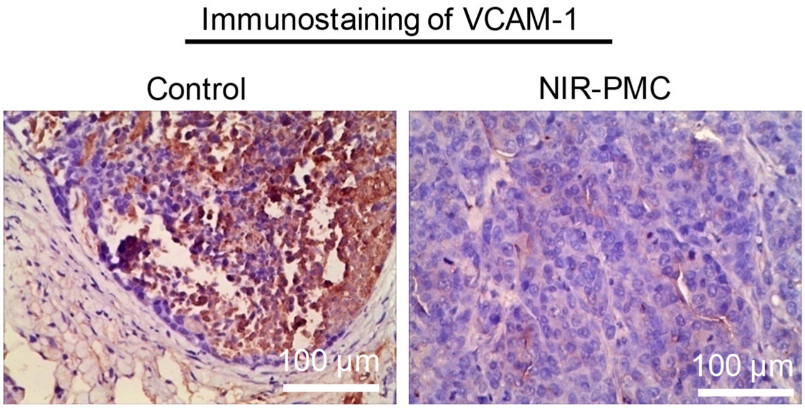

Supplementary Figure 22. Immunostaining of VCAM-1 expression in breast tumors

The tumor and liver samples from rabbits with or without NIR-PMC treatment for 28 d were collected and fixed in 10% formalin for immunohistochemical staining.

(A)

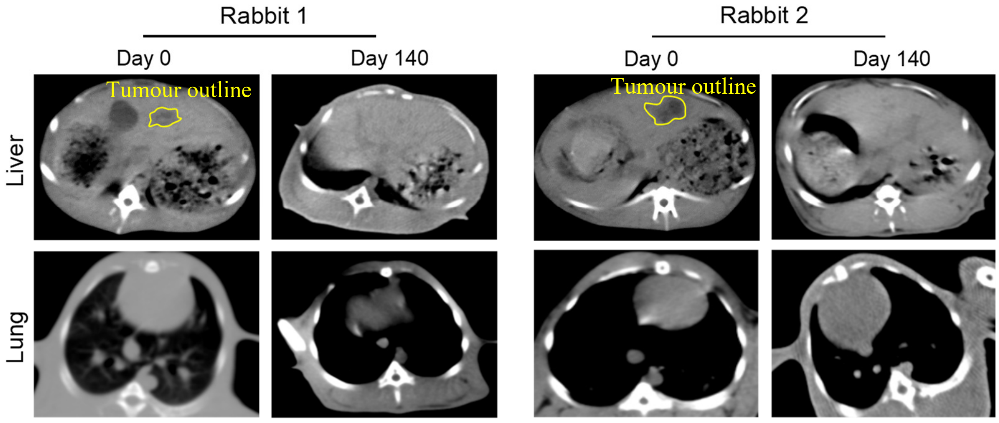

(B)

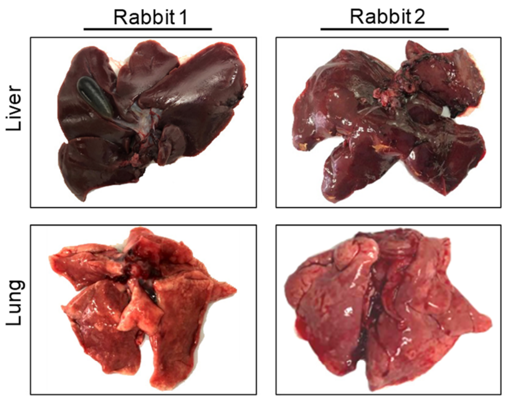

(C)

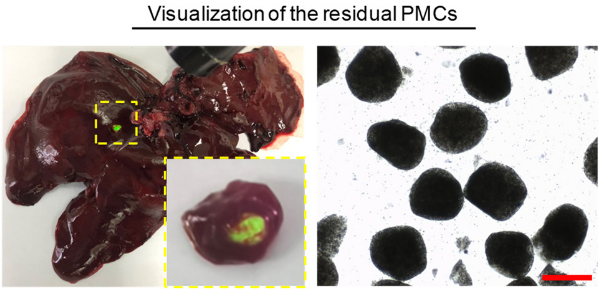

Supplementary Figure 23. CT imaging and *ex vivo* examination of two cancer-free rabbits **(A)** CT images of liver and lungs at 0 and 140 d. The shape/edge of hepatocarcinoma tumors are described by a yellow outline (Scale bars, 1 cm). **(B)** *ex vivo* photos of livers and lungs from the cancer-free rabbits at 140 d. **(C)** Visualization of the residual PMCs by camera and microscopy. The PMCs in livers of tumor-bearing animals at 140 d were imaged by a camera (Canon ZHS2402, Japan). PMCs were retrieved by surgical scissors and suspended in 1 mL PBS buffer for microscopy imaging. Scale bars represent 200 µm.

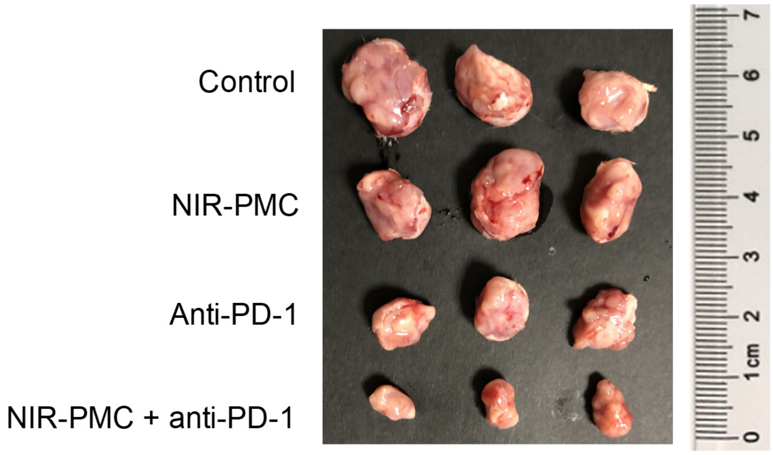

Supplementary Figure 24. Representative photographs of tumors from breast-cancer bearing mice

Animals received different therapeutic agents were sacrificed at 21 d for visualization of orthotopic breast tumors. The *ex vivo* images were captured by a camera (Canon ZHS2402, Japan).

(A) (B)

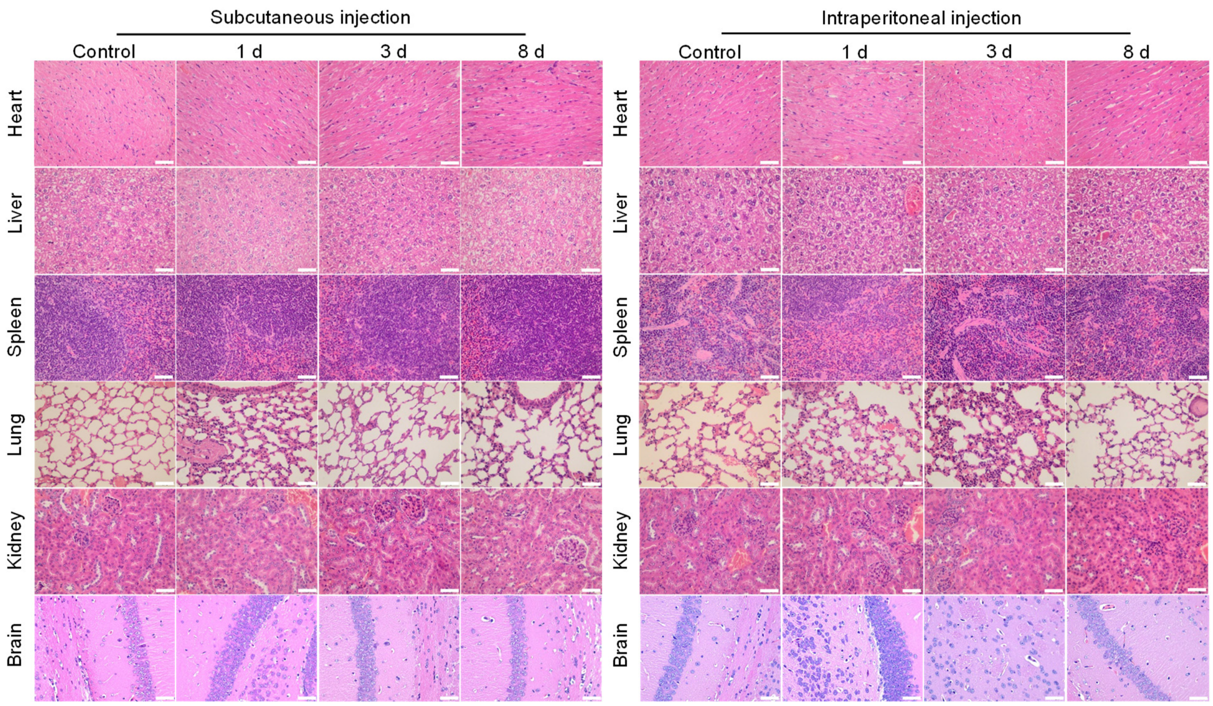

(C)

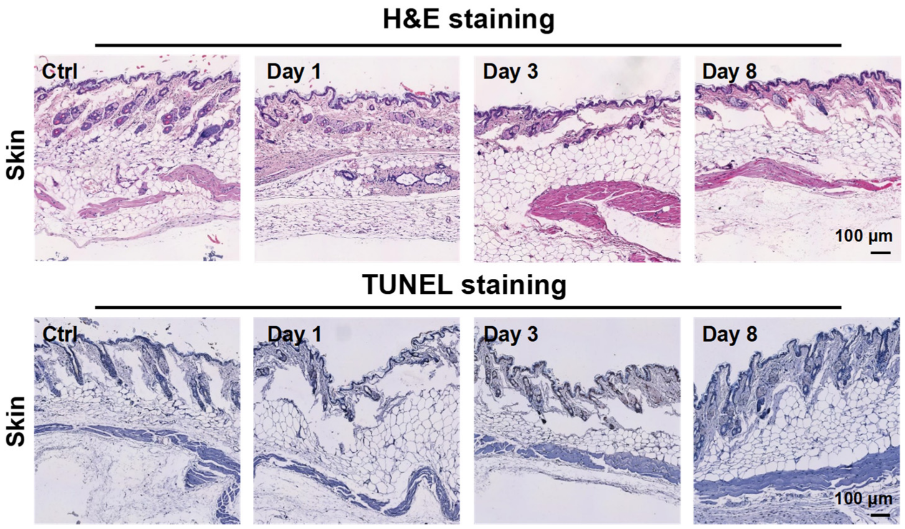

Supplementary Figure 25. Representative histological H&E and TUNEL staining images of organs in mice

H&E staining for **(A)** subcutaneous and **(B)** intraperitoneal microscopy imaging and **(C)** TUNEL staining after 1st, 3rd and 8th d after injection of 25 μL 36000/mL PMCs. The animals were sacrificed to collect heart, liver, spleen, lung, kidney, brain and skin. The tissue samples were fixed in 10% formalin for H&E staining. Scale bars represent 20 μm and 100 μm.

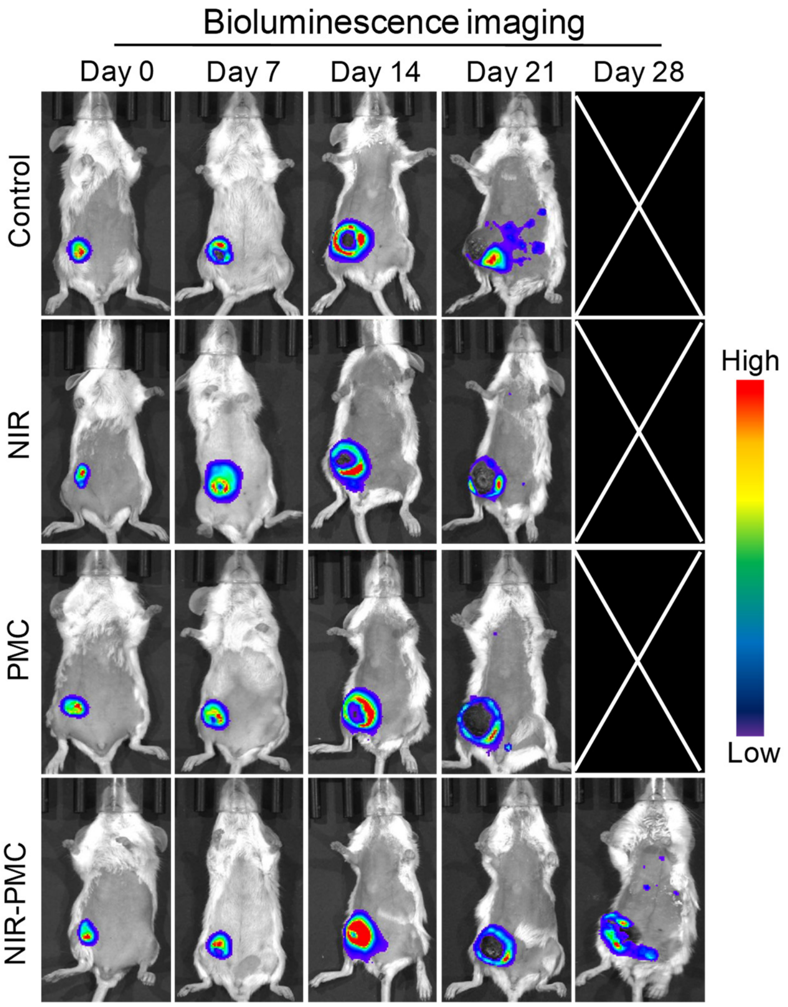

Supplementary Figure 26. Luminescent imaging of breast tumors in mice

Breast-cancer-bearing mice received different therapeutic agents were subjected to intraperitoneal injection of 40 μL D-luciferin solution (40 mg/mL; Biotium, Fremont, CA, USA) prior to imaging by IVIS imaging spectrum system at 0, 7, 14, 21, 28 d.

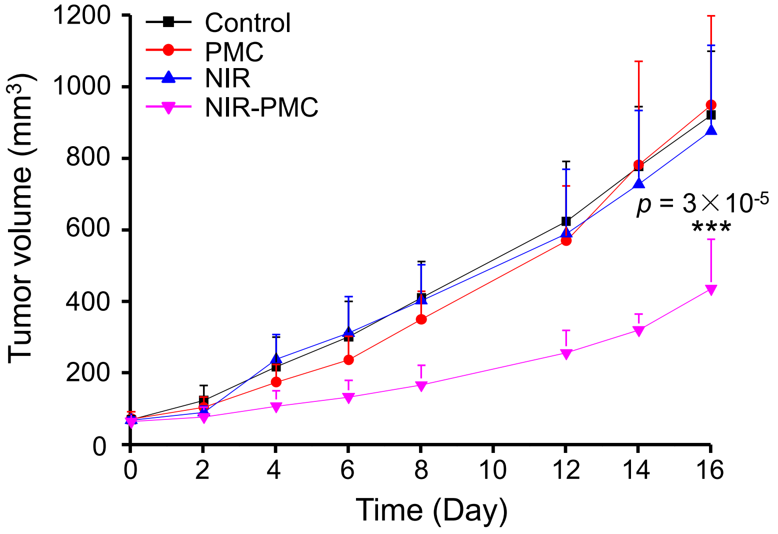

Supplementary Figure 27. Growth curves of breast tumors in mice

The tumor volume of individual mice was measured by vernier caliper every 2 d for 16 d (n = 8). Data are presented as means ± SD. ***$p < 0.001$ compared to control group by two-tailed Student t-test.

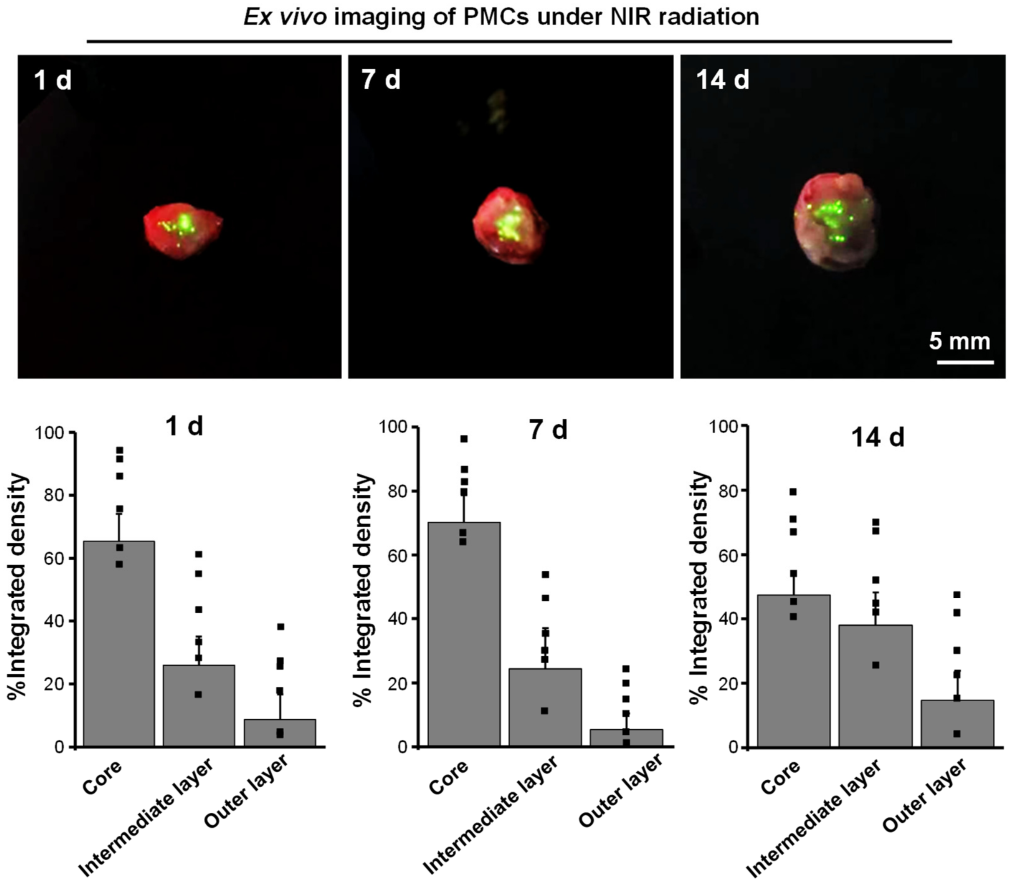

Supplementary Figure 28. Biodistribution of PMCs in tumours

Animals bearing breast tumours with sizes at ~ 5-10 mm were intratumorally injected with 25 µL 36000/mL PMCs or 25 µL PBS (Ctrl). The tumour samples were collected at the 1, 7 and 14 d post injection. After frozen in liquid N_2, the tumour samples were cut at the largest cross section and exposed to 980 nm NIR radiations for visualization. The captured images were subjected to Image J analysis for calculation of the percentage of integrated density in the region of interest. Data are presented as means ± SD, n = 6 image examined over 1 independent experiments.

Western blot images for Supplementary Figure 16

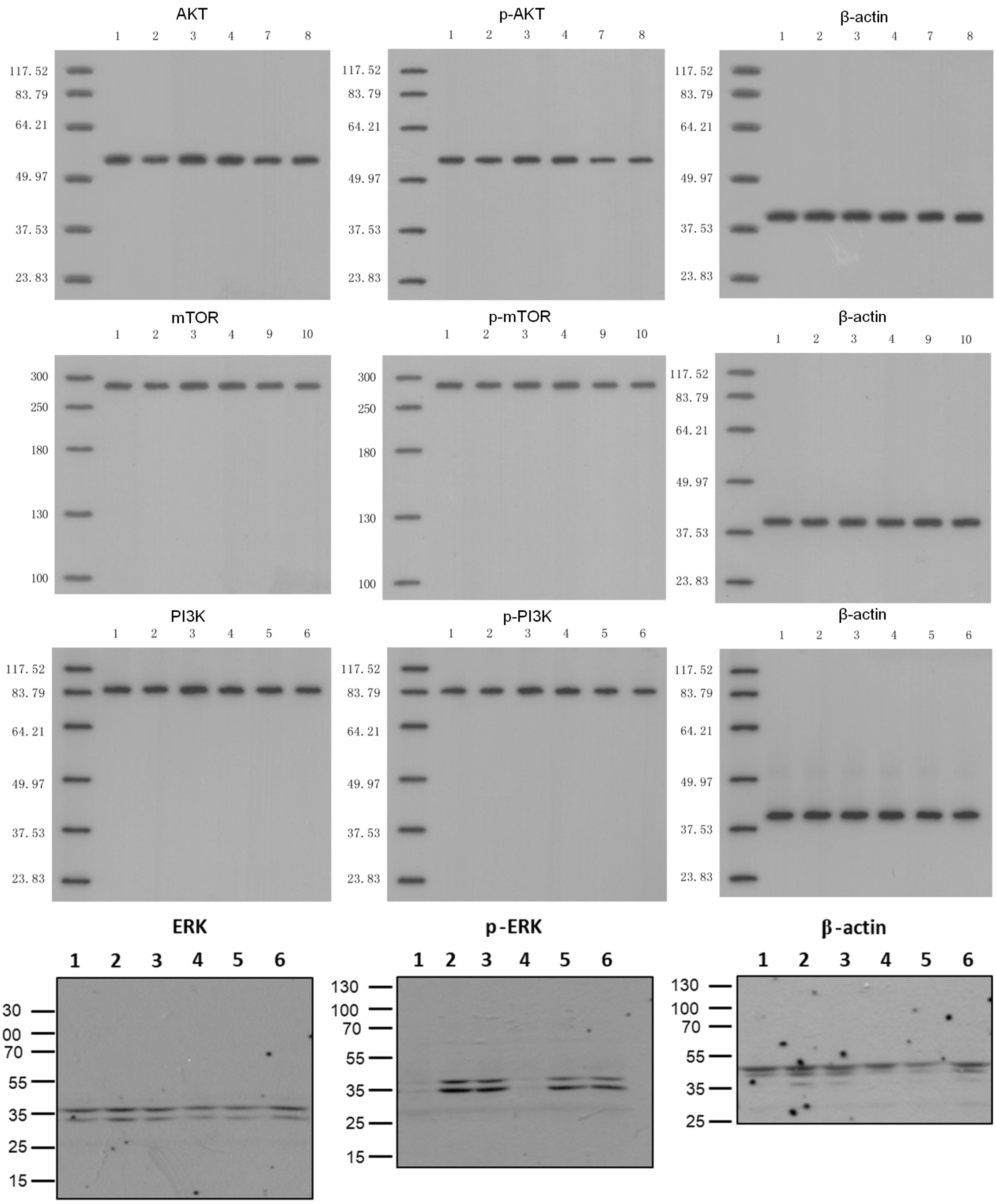

Supplement: Supplementary file 1 — Supplementary Information [file 41467_2022_32066_MOESM1_ESM.pdf]
