# Supplementary material for: Engineering micro oxygen factories to slow tumour progression via hyperoxic microenvironments
Source: Nat Commun. 2022 Aug 2;13:4495. doi: 10.1038/s41467-022-32066-w (PMC9345862; doi:10.1038/s41467-022-32066-w)
Supplement: Supplementary file 2 — Reporting Summary [file 41467_2022_32066_MOESM2_ESM.pdf]

nature portfolio

Corresponding author(s): Ruibin Li

Last updated by author(s): May 8, 2022

Reporting Summary

Nature Portfolio wishes to improve the reproducibility of the work that we publish. This form provides structure for consistency and transparency in reporting. For further information on Nature Portfolio policies, see our Editorial Policies and the Editorial Policy Checklist.

Statistics

For all statistical analyses, confirm that the following items are present in the figure legend, table legend, main text, or Methods section.

n/a	Confirmed	
☐	☒	The exact sample size (*n*) for each experimental group/condition, given as a discrete number and unit of measurement
☐	☒	A statement on whether measurements were taken from distinct samples or whether the same sample was measured repeatedly
☐	☒	The statistical test(s) used AND whether they are one- or two-sided<br>*Only common tests should be described solely by name; describe more complex techniques in the Methods section.*
☒	☐	A description of all covariates tested
☒	☐	A description of any assumptions or corrections, such as tests of normality and adjustment for multiple comparisons
☐	☒	A full description of the statistical parameters including central tendency (e.g. means) or other basic estimates (e.g. regression coefficient) AND variation (e.g. standard deviation) or associated estimates of uncertainty (e.g. confidence intervals)
☐	☒	For null hypothesis testing, the test statistic (e.g. *F*, *t*, *r*) with confidence intervals, effect sizes, degrees of freedom and *P* value noted<br>*Give P values as exact values whenever suitable.*
☒	☐	For Bayesian analysis, information on the choice of priors and Markov chain Monte Carlo settings
☒	☐	For hierarchical and complex designs, identification of the appropriate level for tests and full reporting of outcomes
☒	☐	Estimates of effect sizes (e.g. Cohen's *d*, Pearson's *r*), indicating how they were calculated

Our web collection on statistics for biologists contains articles on many of the points above.

Software and code

Policy information about availability of computer code

Data collection

1. The morphology and size of PMCs were determined by microscopy (FV1200, Olympus, Tokyo, Japan).
2. Photoluminescence spectra of UCNPs were measured with an Edingbour NanoSpectralyzer fluorimetric analyzer (Applied NanoFluorescence, FLS980).
3. A portable dissolved oxygen meter (JPB-70A oxygen sensor, Qiwei Instrument Co., Ltd. Hangzhou, China) was applied to record the dissolved oxygen concentrations.
4. The cell viability data was collected by a Microplate Reader (Synergy NEO HTS, Biotek, USA).
5. Cell imaging were visualized by confocal microscopy (FV1200, Olympus, Japan) .
6. The western-blot assay samples were separated on 8-15% SDS-PAGE gel (Beyotime, Shanghai, China) using tris-glycine-SDS buffer as running buffer in the Mini-PROTEAN Tetra System (Bio-Rad, CA, USA) at 100 V and transferred to a nitrocellulose membrane at 300 mA.The freshly prepared ECL hypersensitive chemiluminescence solution was used to image the membranes by a multi-color fluorescence chemiluminescence imaging analysis system (FluorChem M, Alpha, USA).
7. The LC-MS analysis was performed on an Agilent 1100 Series LC system (Agilent Technologies, Santa Clara, CA, USA) coupled with an LTQXL mass spectrometer (Thermo Fisher Scientific, Waltham, MA, USA).
8. Flow cytometry was performed on FACSVerse, BD Biosciences and data was acquired with the help of FlowJo software 10.6.2(Tree Star Inc.)
9. The PCR analysis was performed in Anhui Leaobei Biotechnology Co. LTD (Tongling, China) by a SYBR Green assay kit (Thermo Fisher, USA) under a Real-Time PCR System (ABI-7300, USA).
10. Bioluminescence imaging of mice was performed with IVIS spectrum in vivo imaging system (PerkinElmer).
11. CT imaging of rabbits were collected by a 16-slice CT spiral scan (Brilliance-16, Phillips, USA) guidance.
12.The tumors were imaged by IVIS imaging spectrum system (PerkinElmer, ME, USA) and Canon camera (Japan).

Data analysis

1. Flow cytometry data were analyzed by FlowJo software 10.6.2 (Tree Star Inc.)
2. MS data was analyzed by Xcalibur software. The concentrations of adenosine in tested samples were determined by its standard curve.

3. qPCR data were analyzed by ABI Prism 7300 SDS Software and the mRNA levels of each gene in two groups were normalized to that of GAPDH. The primers used are listed in Table S2.
4. The blood samples (~400 µL each mouse) were subjected to detection of routine blood indicators by Mindray BC-2800Vet hematology analyzer (Mindray Global, China).
5. Numerical data were statistically analyzed and graphs were generated with the help of Origin85 software and Microsoft excel software.
6. Raw data of animal imaging and bioluminescence imaging were analyzed with the help of Microsoft office software 2010, ImageJ 8.0, and Living image software version 4.5.2 (PerkinElmer)
7. Log rank test were used by SPSS statistics 17.0 software

For manuscripts utilizing custom algorithms or software that are central to the research but not yet described in published literature, software must be made available to editors and reviewers. We strongly encourage code deposition in a community repository (e.g. GitHub). See the Nature Portfolio guidelines for submitting code & software for further information.

Data

Policy information about availability of data

All manuscripts must include a data availability statement. This statement should provide the following information, where applicable:

- Accession codes, unique identifiers, or web links for publicly available datasets
- A description of any restrictions on data availability
- For clinical datasets or third party data, please ensure that the statement adheres to our policy

The datasets generated and/or analyzed during the current study are available in the Zenodo Dataverse repository, by following DOI: 10.5281/zenodo.6588765 https://zenodo.org/record/6588765#.YpqPu8iP-rD

Field-specific reporting

Please select the one below that is the best fit for your research. If you are not sure, read the appropriate sections before making your selection.

[x] Life sciences [ ] Behavioural & social sciences [ ] Ecological, evolutionary & environmental sciences

For a reference copy of the document with all sections, see nature.com/documents/nr-reporting-summary-flat.pdf

Life sciences study design

All studies must disclose on these points even when the disclosure is negative.

Sample size	Mead's resource equation was used to determine the animal numbers in most experiments. Mouse and Rabbit numbers in survival rate experiment were determined by an equation for dichotomous variables (National Journal of Physiology, Pharmacy and Pharmacology 2011;1:35-39). The number of cell samples were determined on the preliminary test results as well as the minimal requirements in Student T-test.
Data exclusions	For in vivo studies, pre-established criteria for animal omission were: 1) failure to inject desired PMC dose and 2) health concerns unrelated to the procedure. In the actual experiments, no animals met these criteria. So, there is no animal exclusion in this study.
Replication	1. Characterization experiments for UCNP nanomaterials: five replicates (Fig. S4);<br>2. Measurements of the partial pressures of oxygen (pO2) in tumours: three replicates (Fig. 2e);<br>3. Characterization and Oxygen product of PMCs: three independent replicates (Fig. S7);<br>4. Quantification of dissolved oxygen in cell culture media: three replicates (Fig. S9);<br>5. Impacts of pressures on oxygenation capability of PMCs: three independent replicates (Fig. S10);<br>6. Impacts of NIR radiation on cell viability: four replicates (Fig. S11);<br>7. Cancer cell proliferation test: six replicates (Fig. 3a);<br>8. Impacts of integrates in PMCs: six replicates (Fig. 3b, Fig. S13);<br>9. Confocal image of newly born cells test: three replicates (Fig. 3c, Fig. S17);<br>10. Western-blot assay: three biologically independent replicates (Fig. 3d);<br>11. Cytotoxicity assessment and cytokine experiments in mammalian cells: three to six replicates (Fig. S12, Fig. 4b, Fig. 4c, Fig S20 and Fig. 6b);<br>12. Detection of apoptotic cells by flow cytometry: three biologically independent replicates (Fig. 4d, Fig. 4e and Fig. S15);<br>13. Impacts of hyperoxia on the cytolytic activity of NK cells: three replicates (Fig. 4f);<br>14. Animal imaging experiments: three replicates (Fig. 5c, Fig. 5e, Fig. 6d);<br>15. Histology experiment: three replicates (Fig. 6a);<br>16. Animal survival rate: ten replicates (Fig. 5g and Fig. 6g);
Randomization	All the cell and animal experiments were randomly allocated into experimental groups by throwing dice.
Blinding	Blind trials were conducted in several critical experiments, including Impacts of NIR-PMCs on cancer cell proliferation, biosafety assessment and animal experiments (e.g. In vivo and ex vivo images, Click-iT®EdU-594 assay, Western-blot assay, HypoxyprobeTM-1 Plus Kit, H&E staining, immunohistochemical staining, tumor growth curves and survival rate). Blinding was not available in some experiments and studies because the operator could easily identify the structure by characteristic signals (TEM, Microscopy images of algae, Absorption spectra of Chlorophyll-α and emission spectra of UCNPs).

Behavioural & social sciences study design

All studies must disclose on these points even when the disclosure is negative.

Study description	*Briefly describe the study type including whether data are quantitative, qualitative, or mixed-methods (e.g. qualitative cross-sectional, quantitative experimental, mixed-methods case study).*
Research sample	*State the research sample (e.g. Harvard university undergraduates, villagers in rural India) and provide relevant demographic information (e.g. age, sex) and indicate whether the sample is representative. Provide a rationale for the study sample chosen. For studies involving existing datasets, please describe the dataset and source.*
Sampling strategy	*Describe the sampling procedure (e.g. random, snowball, stratified, convenience). Describe the statistical methods that were used to predetermine sample size OR if no sample-size calculation was performed, describe how sample sizes were chosen and provide a rationale for why these sample sizes are sufficient. For qualitative data, please indicate whether data saturation was considered, and what criteria were used to decide that no further sampling was needed.*
Data collection	*Provide details about the data collection procedure, including the instruments or devices used to record the data (e.g. pen and paper, computer, eye tracker, video or audio equipment) whether anyone was present besides the participant(s) and the researcher, and whether the researcher was blind to experimental condition and/or the study hypothesis during data collection.*
Timing	*Indicate the start and stop dates of data collection. If there is a gap between collection periods, state the dates for each sample cohort.*
Data exclusions	*If no data were excluded from the analyses, state so OR if data were excluded, provide the exact number of exclusions and the rationale behind them, indicating whether exclusion criteria were pre-established.*
Non-participation	*State how many participants dropped out/declined participation and the reason(s) given OR provide response rate OR state that no participants dropped out/declined participation.*
Randomization	*If participants were not allocated into experimental groups, state so OR describe how participants were allocated to groups, and if allocation was not random, describe how covariates were controlled.*

Ecological, evolutionary & environmental sciences study design

All studies must disclose on these points even when the disclosure is negative.

Study description	*Briefly describe the study. For quantitative data include treatment factors and interactions, design structure (e.g. factorial, nested, hierarchical), nature and number of experimental units and replicates.*
Research sample	*Describe the research sample (e.g. a group of tagged Passer domesticus, all Stenocereus thurberi within Organ Pipe Cactus National Monument), and provide a rationale for the sample choice. When relevant, describe the organism taxa, source, sex, age range and any manipulations. State what population the sample is meant to represent when applicable. For studies involving existing datasets, describe the data and its source.*
Sampling strategy	*Note the sampling procedure. Describe the statistical methods that were used to predetermine sample size OR if no sample-size calculation was performed, describe how sample sizes were chosen and provide a rationale for why these sample sizes are sufficient.*
Data collection	*Describe the data collection procedure, including who recorded the data and how.*
Timing and spatial scale	*Indicate the start and stop dates of data collection, noting the frequency and periodicity of sampling and providing a rationale for these choices. If there is a gap between collection periods, state the dates for each sample cohort. Specify the spatial scale from which the data are taken*
Data exclusions	*If no data were excluded from the analyses, state so OR if data were excluded, describe the exclusions and the rationale behind them, indicating whether exclusion criteria were pre-established.*
Reproducibility	*Describe the measures taken to verify the reproducibility of experimental findings. For each experiment, note whether any attempts to repeat the experiment failed OR state that all attempts to repeat the experiment were successful.*
Randomization	*Describe how samples/organisms/participants were allocated into groups. If allocation was not random, describe how covariates were controlled. If this is not relevant to your study, explain why.*
Blinding	*Describe the extent of blinding used during data acquisition and analysis. If blinding was not possible, describe why OR explain why blinding was not relevant to your study.*

Did the study involve field work? ☐ Yes ☐ No

Field work, collection and transport

Field conditions	*Describe the study conditions for field work, providing relevant parameters (e.g. temperature, rainfall).*
Location	*State the location of the sampling or experiment, providing relevant parameters (e.g. latitude and longitude, elevation, water depth).*
Access & import/export	*Describe the efforts you have made to access habitats and to collect and import/export your samples in a responsible manner and in compliance with local, national and international laws, noting any permits that were obtained (give the name of the issuing authority, the date of issue, and any identifying information).*
Disturbance	*Describe any disturbance caused by the study and how it was minimized.*

Reporting for specific materials, systems and methods

We require information from authors about some types of materials, experimental systems and methods used in many studies. Here, indicate whether each material, system or method listed is relevant to your study. If you are not sure if a list item applies to your research, read the appropriate section before selecting a response.

Materials & experimental systems

n/a	Involved in the study	
☐	☒	Antibodies
☐	☒	Eukaryotic cell lines
☒	☐	Palaeontology and archaeology
☐	☒	Animals and other organisms
☒	☐	Human research participants
☒	☐	Clinical data
☒	☐	Dual use research of concern

Methods

n/a	Involved in the study	
☒	☐	ChIP-seq
☐	☒	Flow cytometry
☒	☐	MRI-based neuroimaging

Antibodies

Antibodies used

Antibody name (supplier name; catalog number; clone name, applicable; validation, dilutions):
1. Phospho-IκBα (Ser32/36) (5A5) Mouse mAb (Cell Signaling Technology; Cat. 9246; monoclonal, WB, IP, IHC, ChIP, IF, F; WB ; 1:1000)
2. IκBα (L35A5) Mouse mAb (Amino-terminal Antigen) (Cell Signaling Technology; Cat. 4814; monoclonal; WB, IP, IHC, ChIP, IF, F; WB ; 1:1000)
3. Anti-Actin antibody produced in rabbit (Sigma-Aldrich; Cat. A2066; polyclonal; ICH, IF, WB; WB ; 1:5000)
4. in vivo Mab anti-mouse PD-1 (CD279) (Bioxcell; Cat. BE0146; monoclonal; WB, ELISA, IP, IHC, ChIP, IF; SDS-PAGE ; 10 mg/Kg mouse)
5. Mouse Anti-AKT antibody (Bioss; Cat. bsm-33278M; monoclonal; WB, ELISA, ICC; WB ; 1:1000)
6. PI3K p85 alpha Antibody (Affinity Biosciences; Cat. AF6241; polyclonal; WB, IHC, IF/ICC, ELISA; WB ; 1:500)
7. Phospho-PI3K p85 (Tyr458) [Tyr467]/p55 (Tyr199) Antibody (Affinity Biosciences; Cat. AF3242; polyclonal; WB, IHC, IF/ICC, ELISA; WB ; 1:500)
8. Anti-phospho-AKT (Abcam; Cat. Ab38449, polyclonal, WB, IHC-P; WB; 1:1000)
9. Rabbit monoclonal [EPR390(N)] to mTOR (Abcam; Cat. Ab134903; monoclonal; WB; Knockout validated; 1:10000)
10. Rabbit monoclonal [EPR426(2)] to mTOR (phospho S2448) (Abcam; Cat. Ab109268; monoclonal; WB, IHC-P, Dot blot, IHC-Fr; Knockout validated; 1:10000)
11. Rabbit monoclonal [Y72] to ERK1 (Abcam; Cat. Ab32537; monoclonal; WB, IP, IHC-P, Flow Cyt (Intra), ICC/IF; Knockout validated; 1:1000)
12. Rabbit monoclonal [EPR18444] to ERK1 (phospho T202) + ERK2 (phospho T185) (Abcam; Cat. Ab214036; monoclonal; Dot blot, IHC-P, WB, ICC/IF, IP; WB; 1:1000)
13. FITC Rat Anti-Mouse IFN-γ (BD Pharmingen; Cat. 554411; monoclonal; IF, FC; verified using the recombinant cytokine blocking and unlabelled antibody blocking specificity control ; 5 μL/test)
14. Purified anti-mouse CD28 Antibody (Biolegend, Cat. 102102; Monoclonal; FC, IP, IHC-F, Costim; Quality tested by intracellular immunofluorescent staining with flow cytometric analysis ; 1 μg/mL)
15. Purified anti-mouse CD3ε Antibody (Biolegend, Cat. 100302; 145-2C11 Monoclonal; FC, IHC-F, IP, WB, ICC; Validated by IHC-F ; 1μL/1×106 cells)

Validation

Information regarding validation and application of the antibodies used can be found in the manufacturers' websites.

Eukaryotic cell lines

Policy information about cell lines

Cell line source(s)	Synechocystis sp. 6803 (catalog No. FACHB-898), Synechococcus elongates 7942 (catalog No. FACHB-805), Scenedesmus obliquus (catalog No. FACHB-417) and Chlorella ellipsoidea (catalog No. FACHB-40) acquired from the Institute of Hydrobiology, CAS (Wuhan, China). HCT116, HepG2, VX2, MCF-7, PANC-1, MGC-803, 4T-1, HeLa and A549 cells were purchased from ATCC (USA). Yac-1 cells were purchased from National Collection of Authenticated Cell Caltures (Shanghai, China). MEF (mouse embryonic fibroblasts) cells were donated by Dr. Sudan He (Suzhou Institute of Systems Medicine, Chinese Academy of Medical Science, China). Luc-4T1 cells were donated by Dr. Zhimou Yang (Nankai University, Tianjin, China). Primary cardiomyocytes, kidney cells, Mouse T cells and NK cells were acquired from BALB/c mice.
Authentication	STR analysis was performed to authenticate HeLa cell in our study. STR loci are amplified using fluorescently labeled PCR primers that flank the hypervariable regions. The result showed HeLa cell used in our study was matched with HeLa in ATCC. The authentication was last performed at May 27th 2022 by Sangon Biotech (Shanghai, China).
Mycoplasma contamination	The cells were all negative in Mycoplasma test.
Commonly misidentified lines (See ICLAC register)	We double checked our cell lines in version 8.0 of ICLAC. There is no misidentified case report on these cells.

Palaeontology and Archaeology

Specimen provenance	*Provide provenance information for specimens and describe permits that were obtained for the work (including the name of the issuing authority, the date of issue, and any identifying information). Permits should encompass collection and, where applicable, export.*
Specimen deposition	*Indicate where the specimens have been deposited to permit free access by other researchers.*
Dating methods	*If new dates are provided, describe how they were obtained (e.g. collection, storage, sample pretreatment and measurement), where they were obtained (i.e. lab name), the calibration program and the protocol for quality assurance OR state that no new dates are provided.*

☐ Tick this box to confirm that the raw and calibrated dates are available in the paper or in Supplementary Information.

Ethics oversight	*Identify the organization(s) that approved or provided guidance on the study protocol, OR state that no ethical approval or guidance was required and explain why not.*

Note that full information on the approval of the study protocol must also be provided in the manuscript.

Animals and other organisms

Policy information about studies involving animals; ARRIVE guidelines recommended for reporting animal research

Laboratory animals	Female BALB/c mice (6-8 weeks old, ~20 g) were obtained from the Experimental Animal Center of the Gempharmatech Co. Ltd in Nanjing, China. Female New Zealand White rabbits (6 months old, weighing 2-2.5 Kg) were obtained from Qingdao Kangda Rabbit Co., Ltd. (Qingdao, China). All animals were grown in an animal facility under filtered air conditions (22-25 °C), 12 h dark/light cycle, 40-60% humidity in plastic cages with sterilized wood shavings for bedding. All animal experiments were strictly performed under the guidelines of the Chinese Council for Animal Care, approved by the Animal Care Committee of the Laboratory Animals in Soochow University.
Wild animals	No wild animals were used in the study.
Field-collected samples	No field-collected samples were used in the study.
Ethics oversight	All animal experiments were strictly performed under the guidelines of the Chinese Council for Animal Care, approved by the Animal Care Committee of the Laboratory Animals in Soochow University.

Note that full information on the approval of the study protocol must also be provided in the manuscript.

Human research participants

Policy information about studies involving human research participants

Population characteristics	*Describe the covariate-relevant population characteristics of the human research participants (e.g. age, gender, genotypic information, past and current diagnosis and treatment categories). If you filled out the behavioural & social sciences study design questions and have nothing to add here, write "See above."*
Recruitment	*Describe how participants were recruited. Outline any potential self-selection bias or other biases that may be present and how these are likely to impact results.*
Ethics oversight	*Identify the organization(s) that approved the study protocol.*

Note that full information on the approval of the study protocol must also be provided in the manuscript.

Clinical data

Policy information about clinical studies
All manuscripts should comply with the ICMJE guidelines for publication of clinical research and a completed CONSORT checklist must be included with all submissions.

Clinical trial registration	*Provide the trial registration number from ClinicalTrials.gov or an equivalent agency.*
Study protocol	*Note where the full trial protocol can be accessed OR if not available, explain why.*
Data collection	*Describe the settings and locales of data collection, noting the time periods of recruitment and data collection.*
Outcomes	*Describe how you pre-defined primary and secondary outcome measures and how you assessed these measures.*

Dual use research of concern

Policy information about dual use research of concern

Hazards

Could the accidental, deliberate or reckless misuse of agents or technologies generated in the work, or the application of information presented in the manuscript, pose a threat to:

No	Yes	
☒	☐	Public health
☒	☐	National security
☒	☐	Crops and/or livestock
☒	☐	Ecosystems
☒	☐	Any other significant area

Experiments of concern

Does the work involve any of these experiments of concern:

No	Yes	
☒	☐	Demonstrate how to render a vaccine ineffective
☒	☐	Confer resistance to therapeutically useful antibiotics or antiviral agents
☒	☐	Enhance the virulence of a pathogen or render a nonpathogen virulent
☒	☐	Increase transmissibility of a pathogen
☒	☐	Alter the host range of a pathogen
☒	☐	Enable evasion of diagnostic/detection modalities
☒	☐	Enable the weaponization of a biological agent or toxin
☒	☐	Any other potentially harmful combination of experiments and agents

ChIP-seq

Data deposition

☐ Confirm that both raw and final processed data have been deposited in a public database such as GEO.

☐ Confirm that you have deposited or provided access to graph files (e.g. BED files) for the called peaks.

Data access links<br>*May remain private before publication.*	*For "Initial submission" or "Revised version" documents, provide reviewer access links. For your "Final submission" document, provide a link to the deposited data.*
Files in database submission	*Provide a list of all files available in the database submission.*
Genome browser session<br>(e.g. UCSC)	*Provide a link to an anonymized genome browser session for "Initial submission" and "Revised version" documents only, to enable peer review. Write "no longer applicable" for "Final submission" documents.*

Methodology

Replicates	*Describe the experimental replicates, specifying number, type and replicate agreement.*

Sequencing depth

Describe the sequencing depth for each experiment, providing the total number of reads, uniquely mapped reads, length of reads and whether they were paired- or single-end.

Antibodies

Describe the antibodies used for the ChIP-seq experiments; as applicable, provide supplier name, catalog number, clone name, and lot number.

Peak calling parameters

Specify the command line program and parameters used for read mapping and peak calling, including the ChIP, control and index files used.

Data quality

Describe the methods used to ensure data quality in full detail, including how many peaks are at FDR 5% and above 5-fold enrichment.

Software

Describe the software used to collect and analyze the ChIP-seq data. For custom code that has been deposited into a community repository, provide accession details.

Flow Cytometry

Plots

Confirm that:

- [x] The axis labels state the marker and fluorochrome used (e.g. CD4-FITC).
- [x] The axis scales are clearly visible. Include numbers along axes only for bottom left plot of group (a 'group' is an analysis of identical markers).
- [x] All plots are contour plots with outliers or pseudocolor plots.
- [x] A numerical value for number of cells or percentage (with statistics) is provided.

Methodology

Sample preparation

BALB/c mice (age: 6-8 weeks; weight: 20 g) were sacrificed by CO2 inhalation to collect spleens. The spleens were ground in 10 mL 1640 RPMI media (10% fetal bovine serum, 100 U/mL penicillin, 100 µg/mL streptomycin). The mixture was filtered by 200 mesh nylon membrane (Cat. 7061011, Dakewe, China) to remove tissue debris. The suspensions were centrifuged (Allegra 64R, Beckman) at 1300 rpm for 5 min to collect cell pellets, followed by twice washing in PBS. The cell pellets were dispersed and incubated in 5 mL 1× RBC red cell lysis buffer (8.26 g/L NH4Cl, 1g/L KHCO3 and 0.037 g/L EDTA in DI H2O) at room temperature for 5 min. Subsequently, RPMI 1640 media (5 mL) were added to stop the lysis reaction and centrifuged to collect the splenocytes, which were used for expansion of T cells. The splenocytes which were prepared as described above were resuspended in RPMI 1640 media at 2×106/mL, added into the CD28/CD3 antibodies-coated plates (5mL/well) and incubated for 48 h in the presence of 200 IU/mL IL-2 for expansion of T cell. Then the pre-activated T cells were collected and transferred into 24-well plates (2×106/mL, 2 mL/well) for further test of IFN-γ production. Mouse T cells pre-activated with CD3 and CD28 antibodies were resuspended in RPMI-1640 supplemented with 10% FBS, then seeded in the lower chambers of 24-transwell plates (2×106 cells/well, 2 mL) with 50 ng/mL PMA and 1 µL/mL Golgi inhibitor. PMCs were added into the upper chamber (3600/well) with 8.0 µm pore size on PC membranes. Then the whole cultures were exposed to three interval NIR radiation. After 18 h, the cells were spun down at 700 g for 5 min, and the pelleted cells were fixed in BD Cytofix/Cytoperm buffer for 30 min. The fixed cells were washed by BD Perm/Wash buffer (Cat. 554723, BD Biosciences) once, and then the cells were resuspended in BD Perm/Wash buffer containing 5 µL/test anti-IFN-γ-FITC (Cat. 554411, BD Biosciences) for incubation at room temperature for 30 min. After thrice washing in BD Perm/Wash buffer, the resuspended cells were subjected to flow cytometry analysis.

Instrument

The instrument for data collection is FACSVerse, BD

Software

Data analysis was carried out using FlowJo software 10.6.2 (Tree Star Inc.)

Cell population abundance

At least 10,000 total events were acquired for all ananlyses

Gating strategy

In general, cells were first gated by FSC-A/SSC-A to exclude debris including dead cells, then FSC-A/FSC-H to exclude doublets followed by specific antibody stain (e.g., CD3 etc.).

- [x] Tick this box to confirm that a figure exemplifying the gating strategy is provided in the Supplementary Information.

Magnetic resonance imaging

Experimental design

Design type

Indicate task or resting state; event-related or block design.

Design specifications

Specify the number of blocks, trials or experimental units per session and/or subject, and specify the length of each trial or block (if trials are blocked) and interval between trials.

Behavioral performance measures

State number and/or type of variables recorded (e.g. correct button press, response time) and what statistics were used to establish that the subjects were performing the task as expected (e.g. mean, range, and/or standard deviation across subjects).

Acquisition

Imaging type(s) | *Specify: functional, structural, diffusion, perfusion.*

Field strength | *Specify in Tesla*

Sequence & imaging parameters | *Specify the pulse sequence type (gradient echo, spin echo, etc.), imaging type (EPI, spiral, etc.), field of view, matrix size, slice thickness, orientation and TE/TR/flip angle.*

Area of acquisition | *State whether a whole brain scan was used OR define the area of acquisition, describing how the region was determined.*

Diffusion MRI ☐ Used ☐ Not used

Preprocessing

Preprocessing software | *Provide detail on software version and revision number and on specific parameters (model/functions, brain extraction, segmentation, smoothing kernel size, etc.).*

Normalization | *If data were normalized/standardized, describe the approach(es): specify linear or non-linear and define image types used for transformation OR indicate that data were not normalized and explain rationale for lack of normalization.*

Normalization template | *Describe the template used for normalization/transformation, specifying subject space or group standardized space (e.g. original Talairach, MNI305, ICBM152) OR indicate that the data were not normalized.*

Noise and artifact removal | *Describe your procedure(s) for artifact and structured noise removal, specifying motion parameters, tissue signals and physiological signals (heart rate, respiration).*

Volume censoring | *Define your software and/or method and criteria for volume censoring, and state the extent of such censoring.*

Statistical modeling & inference

Model type and settings | *Specify type (mass univariate, multivariate, RSA, predictive, etc.) and describe essential details of the model at the first and second levels (e.g. fixed, random or mixed effects; drift or auto-correlation).*

Effect(s) tested | *Define precise effect in terms of the task or stimulus conditions instead of psychological concepts and indicate whether ANOVA or factorial designs were used.*

Specify type of analysis: ☐ Whole brain ☐ ROI-based ☐ Both

Statistic type for inference
(See Eklund et al. 2016) | *Specify voxel-wise or cluster-wise and report all relevant parameters for cluster-wise methods.*

Correction | *Describe the type of correction and how it is obtained for multiple comparisons (e.g. FWE, FDR, permutation or Monte Carlo).*

Models & analysis

n/a	Involved in the study
☐	☐ Functional and/or effective connectivity
☐	☐ Graph analysis
☐	☐ Multivariate modeling or predictive analysis

Functional and/or effective connectivity | *Report the measures of dependence used and the model details (e.g. Pearson correlation, partial correlation, mutual information).*

Graph analysis | *Report the dependent variable and connectivity measure, specifying weighted graph or binarized graph, subject- or group-level, and the global and/or node summaries used (e.g. clustering coefficient, efficiency, etc.).*

Multivariate modeling and predictive analysis | *Specify independent variables, features extraction and dimension reduction, model, training and evaluation metrics.*
